# Origin identification of Chinese Maca using electronic nose coupled with GC-MS

**DOI:** 10.1038/s41598-019-47571-0

**Published:** 2019-08-21

**Authors:** Aimin Li, Shenglin Duan, Yanting Dang, Xi Zhang, Kai Xia, Shiwei Liu, Xiaofeng Han, Jian Wen, Zijie Li, Xi Wang, Jia Liu, Peng Yuan, Xiao-Dong Gao

**Affiliations:** 1 Key Laboratory of Carbohydrate Chemistry and Biotechnology, Ministry of Education, School of Biotechnology, Jiangnan University, Wuxi, 214122 China; 2 Beijing Key Laboratory of the Innovative Development of Functional Staple and Nutritional Intervention for Chronic Diseases, China National Research Institute of Food and Fermentation Industries Co., LTD., Beijing, 100015 China; 3 Analytical Instruments Dept. Analytical Application Center Shimadzu Co., LTD, Beijing, 100020 China

**Keywords:** Plant sciences, Medical research

## Abstract

Maca (*Lepidium meyenii* Walp.), originated in the high Andes of Peru, is rich in nutrients and phytochemicals. As a new resource food in China, Maca suffers marketing disorders due to the limitation of basic research. Due to the close relationship of Maca quality and origin of place, it’s of scientific, economic and social importance to set up a rapid, reliable and efficient method to identify Maca origin. In the present study, 303 Maca samples were collected from 101 villages of the main producing area in China. Using electronic nose and BP neutral network algorithm, a Maca odor database was set up to trace the origin. GC-MS was then employed to analyze the characteristic components qualitatively and semi-quantitatively. As a result, very significant differences (*p* < 0.01) were detected in the volatile components of Maca from different areas. This study not only constructs a network model to forecast the Maca origin, but also reveals the relationship between Maca odor fingerprints and origins.

## Introduction

Maca (*Lepidium meyenii* Walp., *Brassicaceae*) is an annual and biannual herb plant, and is originated from the Andes of Peru with an attitude of 3500 m and higher^1–3^. Due to the rich nutrients, Maca is widely used to improve health^4^. The bioactive components of Maca include Macaenes^5^, isothiocyanate^6^, sterol^7^, phenolic compounds^8^, secondary metabolites and derivatives^9^, glucosinolates^10,11^, etc. These components have multiple pharmacological functions including sexual and fertility enhancement, anti-fatigue, anti-oxidization, and so on^12^. Moreover, natural compounds lepidiline A and B from Maca exhibit potent cytotoxic activity against several human cancer cell lines^13^. By synthesizing a series of hybridizing compounds of carbazole with imidazole moieties, the antitumor activity of carbazole imidazolium salt derivatives have been tested, and substitution of the imidazolyl-3-position with a 2-bromobenzyl or naphthylacyl group as well as alkyl chain length between carbazole and imidazole ring were found to be important for the anti-tumor activity of cell lines HL-60, SMMC-7721, MCF-7 and SW480 by inducing cell cycle arrest and apoptosis^14^.

In Peru, Maca is called “ginger” and has been taken 5800 years ago. In the 1980s, international organizations like FAO promote the Maca production worldwide for health purposes. Later on, Maca as a functional food became popular in America, Europe, Japan, and so on. In 1992, Maca was listed as one of the rare plants by the International Plant Genetic Resources. The wide use of Maca in China is relatively new. In 2002, Maca was imported into China under the administration of Ministry of Health. In 2003, Maca was successfully transplanted in Lijiang, Yunnan. In 2011, Maca powder was registered as a new resource food in the Department of Health Planning of China. From then on, the Maca business has been rapidly developing. Until 2014, the international Maca market achieved 10 billion USD, while China market accounted for one sixth^15^.

Maca grows in high-altitude alpine areas. It has been reported that the origin of Maca is the key factor to determine the type and accumulation of secondary metabolites, that is, the quality of Maca and its products depends on the geographic origin^16–18^. Although planting history and Maca phenotype also influence the composition of secondary matobolites^6,8^, Maca origin is still the most important factor. However, along with the increasing requirements, the Maca market is suffering disorders like seconds sold in the best price and spurious ones mixed with the genuine. Without the characteristic chemical database of Maca, quality control is hard to be executed. Thus it is of importance to establish a reliable and efficient method to identify Maca origin.

Maca smells special, and the types and compositions of Maca volatiles might be characteristic. In this study, electronic nose was used to collect the odor fingerprints of 303 Maca samples of the main producing area. By using the pattern recognition toolbox, a BP neural network was constructed to identify the Maca origin, and its performance was tested against a few sets. Further GC-MS analysis identified the volatile types and compositions of different Maca samples. The microscopic and macroscopic results altogether revealed the correlation between Maca odor fingerprints and volatiles. The mass identification of Maca samples at low cost will solve the fake and inferior problems of the Maca market, and provides scientific support to standardize the Maca industry.

## Results and Discussion

### Maca sampling and powder preparation

There are many factors, such as planting conditions, place of origin, drying method, color type, that influence the metabolites of Maca^2,5,6,17,19,20^. In the present study, a total of 303 Maca samples were collected from 101 villages, 19 towns, six cities and two provinces of the main producing area in China (Table S1). These samples had similar planting cycles, i.e. breeding in greenhouse in March to April, transplanting to fields in June, and being harvested in December or the next January and February. Because Maca grew at high altitude, no fertilizer, pesticide or weeding was applied, and therefore the growth conditions are close to the wildness. Moreover, all samples were fresh roots, and were pretreated using the same method. Since color types have little effects on Maca^5,20,21^, the origin of Maca was determined to be the only key factor related to the type and concentration of Maca metabolites.

### Selection and optimization of the electronic nose parameters

To meet the standards of electronic nose for better accuracy and efficiency, the parameters were set as follows: carrier, high purity air; sampling time, 120 s; delay time of data acquisition, 18 min; loading amount, 4 mL; loading rate, 1 mL/s; sampler incubation time, 120 s; sampler incubation temperature, 50 °C; and syringe temperature, 60 °C. Under the optimized conditions, representatives of Maca samples released the strongest signals (Fig. S1). The instrument stability was also assessed against 70 Maca samples. The data collected by ten odor detectors all showed a relative standard deviation of less than 15% (Table S2). It indicates that the odor detection system is accurate, efficient and reliable. Using this system, the odor fingerprint of each Maca sample was produced by the electronic nose equipped with 17 sensors (Table S3). In comparison to previous Maca studies that scanned a large area across different countries or different regions of the same country or focused on one or several specific metabolites^5,11,22,23^, this study collected the odor data of 303 Maca samples of two neighboring Maca-producing provinces and would provide more valuable and systematic information for the study of Maca.

### BP neural network of Maca odors

**Figure 1:**
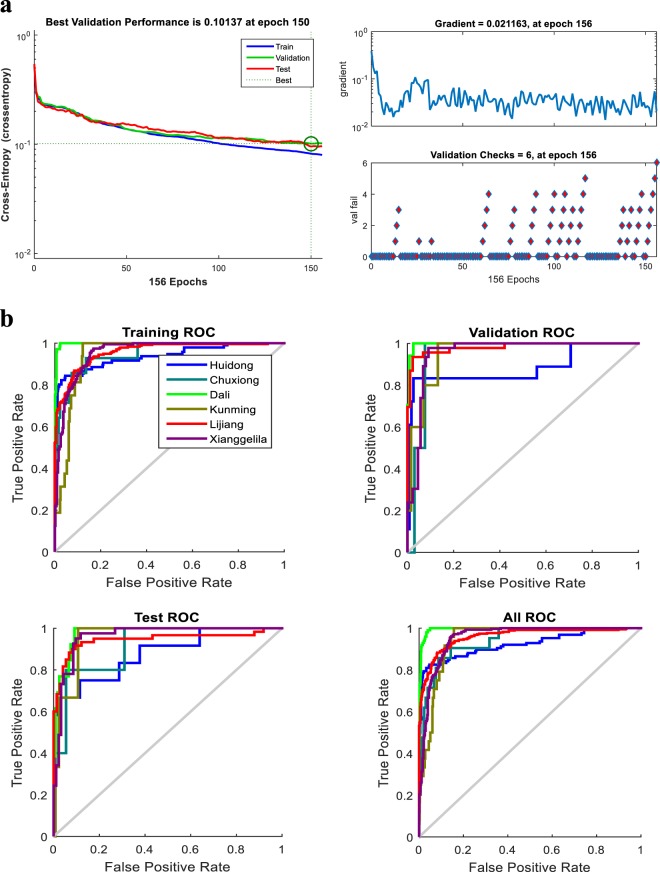
Construction of the BP neural network. (**a**) Performance of the training set. (**b**) ROC curve of the training set.

A two-level BP neural network was constructed to identify the Maca origins (Table 1) based on their odor fingerprints. The level 1 (cities) consisted of an input layer of 17 neurons, a hidden layer of 15 neurons, and an output layer of 6 neurons. The level 2 (towns) was subordinate to the level 1, in which the output layer of Huidong (the only city containing six towns) had 6 neurons. Cross-entropy cost function was used to test the performance of the BP neural network. When the iteration occurred 150 times, the cross-entropy was down to 0.10137, and the signals were transferred back to the input layer. Further optimization was conducted using the conjugate gradient descent algorithm. With 156 times of iterations, the gradient was decreased to 0.21163, and the best network was constructed (Fig. 1a). Receiver operating characteristic curve (ROC) was used to assess the performance of the BP neural network. The larger the area under curve (AUC), the better the BP neural network (Fig. 1b). After optimization, the AUC values of the six cities were all higher than 0.8. It indicated that the BP neural network we constructed is reliable for Maca origin prediction.

**Table 1:** The two-level BP neural network used to identify Maca origins.

Province	City (Level 1)	Town (Level 2)	No. of sample
Yunnan	Chuxiong	Donghua	5
	Dali	Eryuan	5
		Heqing	25
		Jianchuan	3
	Kunming	Dongchuan	1
		Luquan	8
	Lijiang	Yulong	81
		Ninglang	36
		Yongsheng	2
	Shangri-La	Weixi	24
		Hutiaoxia	18
		Jiantang	14
		Xiaozhongdian	36
Sichuan	Huidong	Baishan	4
		Manyingou	17
		Duge	2
		Wudongde	1
		Qianxin	9
		Lama	12
Total	6	19	303

**Figure 2:**
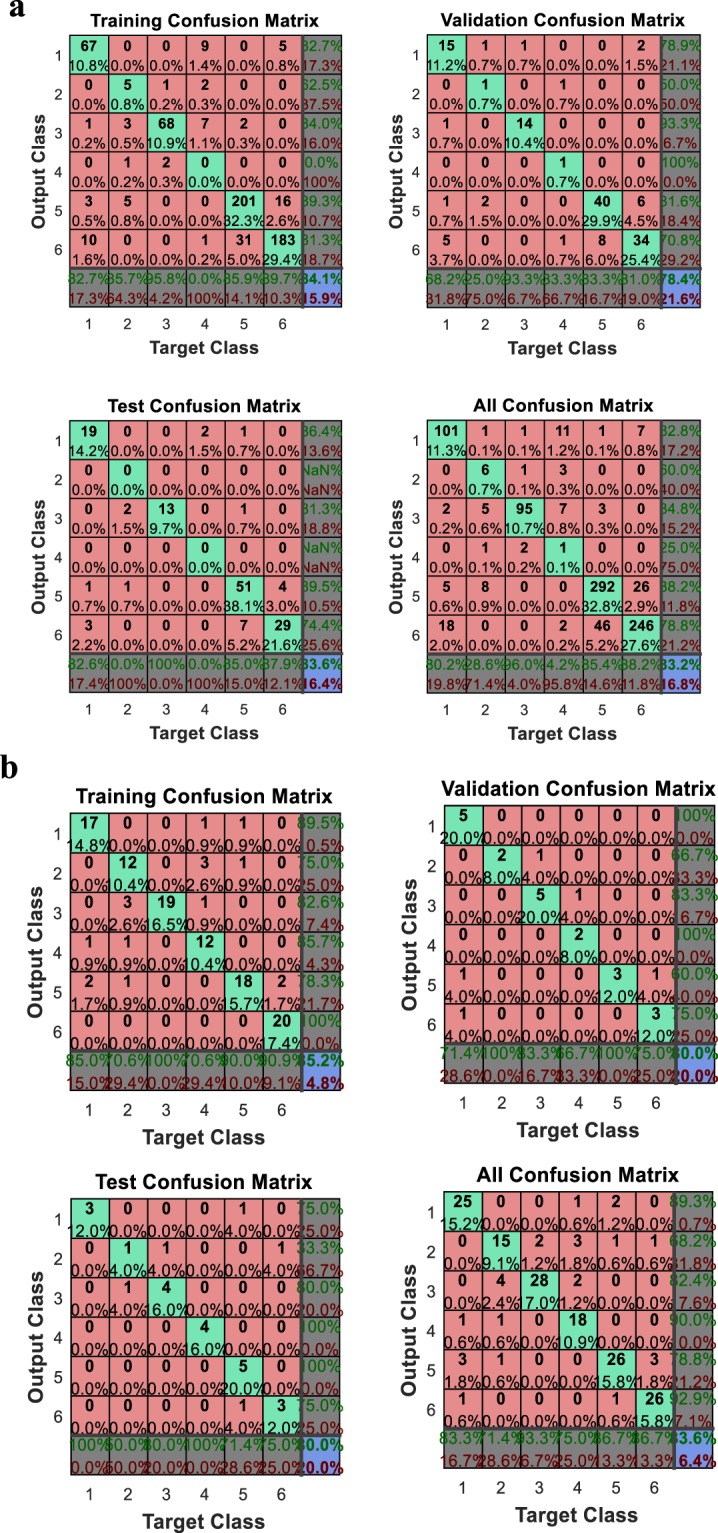
Confusion matrix to evaluate the prediction accuracy of 293 Maca samples (**a**) and 239 samples after weighted treatment (**b**) of six cities. The classes are: 1, Xichang; 2, Chuxiong; 3, Dali; 4, Kunming; 5, Lijing; and 6, Shangri-La.

**Figure 3:**
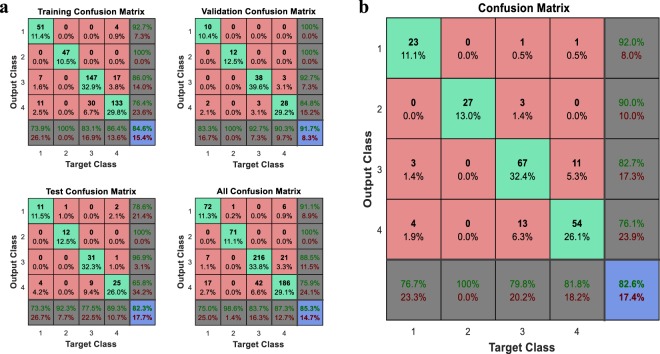
Confusion matrix of 211 Maca training samples (**a**) and 72 test samples (**b**) of four cities. The classes are: 1, Xichang; 2, Dali; 3, Lijing; and 4, Shangri-La.

In the food industry, a trained BP neural network with a correct forecast rate of more than 70% is recognized to be reliable and efficient^24^. In this study, we used confusion matrix to evaluate the predictability of BP neural network. For initial test, 297 Maca samples of six cities (Xichang, Chuxiong, Dali, Kunming, Lijiang and Shangri-La assigned to number 1 to 6) were subjected to the analysis, and the corresponding output values for training, validation, test and overall were 84.1%, 78.4%, 83.6%, and 83.2% respectively (Fig. 2a). Due to the small area of Maca plantation in Kunming and Chuxiong, fewer samples were collected, which caused low recall and inaccurate prediction inaccuracy of these areas. By decreasing the sample sizes of other cities^16^, the weights of Chuxiong and Kunming were improved, with the increased recall rates of 71.4% and 75.0%, and the recognition accuracy was improved to 83.6% (Fig. 2b). To predict the majority accurately, Chuxiong and Kunming were excluded, and Maca samples of the other four cities were randomly selected for training (211 samples) and test (72 samples) of the BP neuron network. As a result, 85.3% of training samples (Fig. 3a) and 82.6% of test samples (Fig. 3b) were predicted to be correct. It indicated that the level 1 of BP neural network is reliable to predict the Maca origin.

The level 2 network (Huidong, the only city containing six towns) was constructed. After 36 times of iteration, the minimum cross-entropy (0.041636) was achieved. Six more iterations had no improvement, and the best network was then constructed. ROC analysis indicated that the level 2 BP neuron network performed well, as each town had an AUC value of close to 1.0. To improve the accuracy and applicability of the network, samples from Wudongde were excluded (with an AUC value of 0). When assigned Baishan, Duge, Lama, Manyingou, Qianxin and Wudongde to number 1 to 6, the accurate rates of training, validation, testing, and overall were 83.8%, 92.9%, 93.0%, and 86.5%, respectively. Using the level 2 BP neuron network of Huidong, the origin of 81.8% samples was accurately predicted.

Using the main Maca-producing area of China as a large sampling site, the Maca odor fingerprints were collected by electronic nose, and a BP neural network of two levels based on Maca odor was constructed to differentiate Maca of different origins (down to towns). By retaining most of the natural ingredients^25^, this method is simple, rapid, and accurate to identify a large number of Maca samples across low geographic span and of different phenotypes, and thus is applicable for quality control in industries and markets.

### Identification of the Maca volatiles by GC-MS

In this study, we used SPME-GC/MS to analyze the Maca volatiles. The performance of three solid microectractors (85 μm PA, 95 μm CAR WR/PDMS, and 75 μm CAR/PDMS) was compared using the Maca sample YXHD-1B. Qualitative and semi-quantitative analysis indicated that these microextractors varied in the detection of sorts and contents of Maca volatiles. Of them, CAR/PDMS having capability of detecting 80 chemicals (vs. 76 and 72 chemicals) and a total content of 671.2 ng/mg (vs. 101.8 and 128.7 ng/mg) was selected for further GC/MS analysis.

With reference to standard chromatographs and literatures^26,27^, 81 volatiles of ester, acid, ketone, aldehyde, alkane, olefin, indole, alcohol, aromatic, pyridine, pyrazine, furan, pyrrole, heterocycle, amide, ether, and pyrimidine (Table S4) were detected in 95 Maca samples of 8 towns. Of them, 53 compounds were common, and 28 were specific.

**Figure 4:**
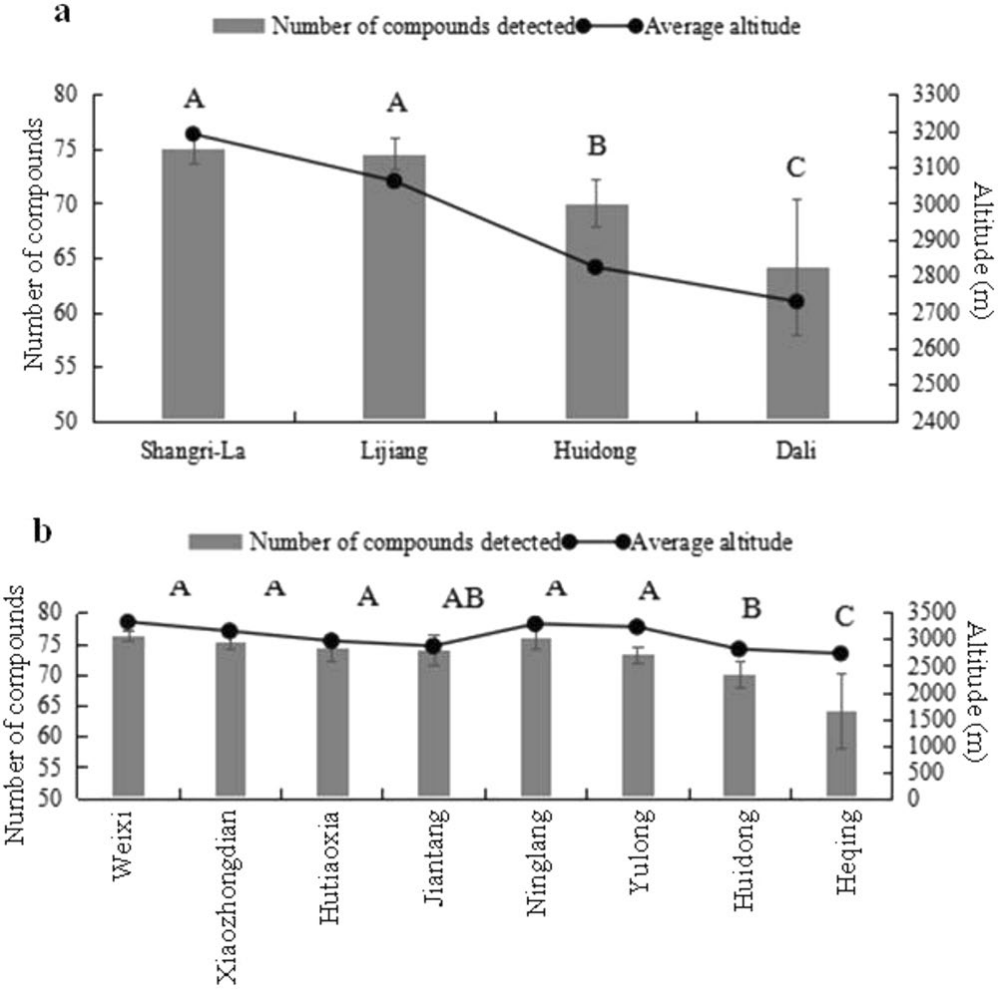
Number of compounds detected in Maca samples of different altitudes. (**a**) Different cities. (**b**) Different towns.

The number of compounds of each Maca sample was then subjected to one-way ANOVA analysis. As shown in Fig. 4, the compound numbers of Maca samples from Shangri-La and Lijiang were significantly higher (*p* < 0.01) than that of Xichang and Dali. With towns as the units, the Maca samples from Weixi, Xiaozhongdian, Hutiaoxia, Ninglang, and Yulong had more compound numbers detected than those from Huidong and Heqing (*p* < 0.01). Further analysis indicated a correlation between the compound number and sampling altitude, i.e. more compounds were detected in the high-altitude area of Shangri-La and Lijiang.

To distinguish different Maca origins, the compounds only detected in a specific area and surroundings were defined as characteristic. As shown in Table 2, 4-methyl-tridecane in Heqing, n-hydroxy methyl-2-phenylacetamide in Jiantang, nonanal in Jiantang and Xiaozhongdian, benzoic acid in Huidong and Heqing, 6-hydroxy-4(1H)-pyrimidinone in Hutiaoxia and Weixi, and p-xylene in Ninglang, Yulong, Weixi and Xiaozhongdian were only detected and treated as characteristic compounds.

**Table 2:** Characteristic compounds of Maca from different origins.

Compound	Huidong	Heqing	Ninglang	Yulong	Hutiaoxia	Jiantang	Weixi	Xiaozhongdian
Nonanal	−	−	−	−	−	+	−	+
4-Methyl-tridecane	−	+	−	−	−	−	−	−
Benzoic acid	+	+	−	−	−	−	−	−
N-Hydroxy methyl-2-phenylacetamide	−	−	−	−	−	+	−	−
p-Xylene	−	−	+	+	−	−	+	+
6-Hydroxy-4(1H)-pyrimidinone	−	−	−	−	+	−	+	−

+, detected; −, undetected.

### PLS-DA analysis of the Maca volatiles

In previous studies, some Maca metabolites have been used to identify Maca origin. For example, the Maca samples from Junin and Ancash were differentiated based on the contents of glucosinolates^18,28,29^. By combining the Maca characteristic fingerprints and PLS-DA analysis, the Maca samples from the northeast of Yunnan, the northwest of Yunnan, and Peru were successfully separated^30^. However, these studies are limited by further identification into lower levels.

**Figure 5:**
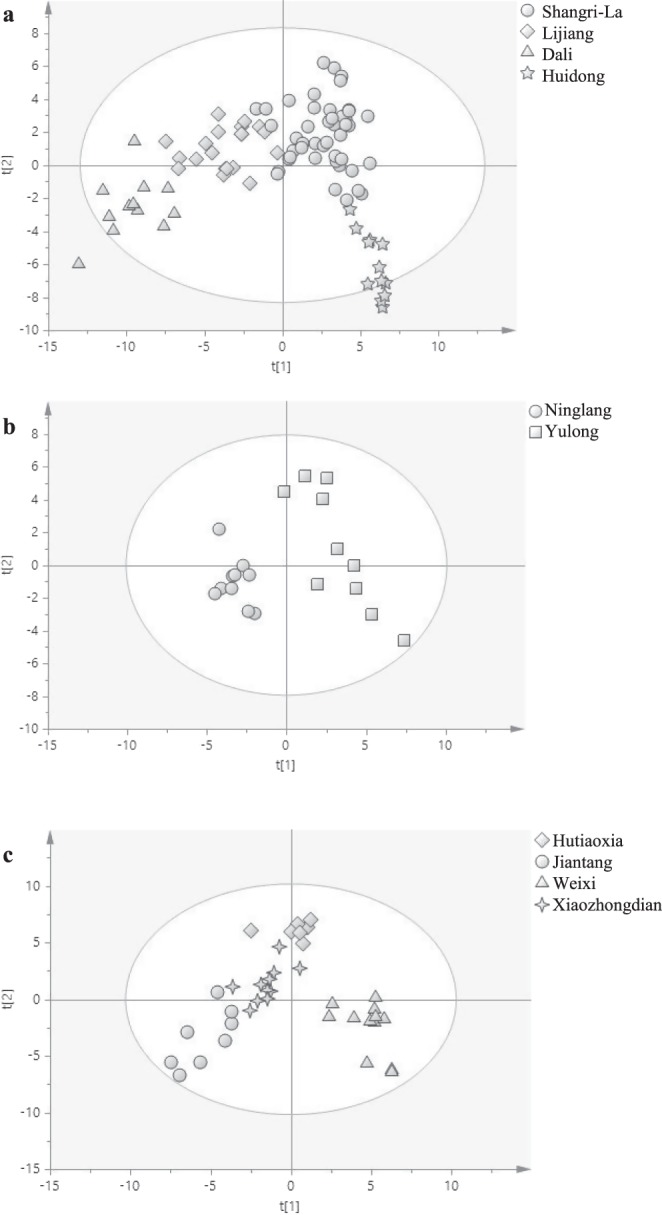
PLS-DA analysis of the Maca samples collected from different cities (**a**) and towns Ninglang and Yulong of Lijiang (**b**) and Weixi, Hutiaoxia, Jiantang and Xiaozhongdian of Shangri-La (**c**).

In this study, a PLS-DA model was built to distinguish regions of Maca. based on the data of volatile compounds of Maca (Table S4). As shown in Fig. 5, samples from the same cities congested together and kept distant from other samples. Some samples from Dali and Huidong had different concentrations of volatiles, and were distributed out of the 95% confidence interval. The R^2^X(cum) and R^2^Y(cum) values of 0.781 and 0.696 indicated that samples of different origins varied both in the compound types and concentrations. The accuracy (Q^2^) was high up to 95.8%, suggesting this model is applicable in the prediction of Maca origin. The VIP of each compound was then calculated from the PLS-DA model to analyze the contribution of each variant^27^. Forty-three volatiles with a VIP value of >1 showed great variances among different Maca samples, which followed the order of eugenol (1.8252) >sunflower aldehyde (1.4364) > ethylbenzene (1.4038) > γ-octalactone (1.3727) > octanol (1.3646).

PLS-DA models of level 2 were also constructed with the 85 compounds as variant X and the towns Ninglang and Yulong of Lijiang or the Weixi, Hutiaoxia, Jiantang and Xiaozhongdian of Shangri-La as variant Y. In Lijiang, samples from Ninglang and Yulong separated distinctively, and those from Yulong were relatively dispersed. It might be ascribed to the larger longitude scan of Yulong samples. In Shangri-La, samples from Weixi were separated distinctively from Hutiaoxia, Jiantang and Xiaozhongdian. The R^2^X(cum) and R^2^Y(cum) values of the PLS-DA models of Lijiang and Shangri-La were 0.981 and 0.865 and 0.940 and 0.807, respectively, and the predicting accuracy (Q^2^) was both 100.0%. It indicates that Maca samples from different towns are distinct in both chemical types and concentrations. The models are applicable to predict the origin of most Maca samples. Thirty-one and 38 volatiles collected from Lijiang and Shangri-La with a VIP value of > 1 showed great variances among different Maca samples. The most important five chemicals were sunflower aldehyde (2.083) > acetic acid (2.0317) > chlorobenzene (1.8584) > isopropyl acetone (1.7155) > 2-methyl butyric acid (1.7150) in Lijiang, and ethyl acetate (1.5122) > acetic acid (1.4434) > geosmin (1.3856) > 2-methyl butyric acid (1.3780) > benzyl alcohol (1.3322) in Shangri-La, respectively. These compounds might be used for differentiation of Maca from different origins.

### Correlation analysis

The relationship of 17 sensors of electronic nose and 81 Maca volatiles was also determined. As shown in Table S3, the sensors varied in the responses to different types and concentrations of chemicals, corresponding to the mechanism of electronic nose^31^. Of them, sensor LY2/LG was sensitive to 2-hexanone, caprolactam, n-valeric acid, hexanal, caproic acid, 2-isobutyl-3-methoxy pyrazine, lauric acid, and 1-pentanol, while sensors PA/2 and P30/2 were only sensitive to geraniol and methyl salicylate, respectively. These correlations were significant (*p* < 0.05). Therefore, through correlation analysis of the odor fingerprints and volatiles of Maca, characteristic chemicals or chemicals with great variance were identified in the Maca samples of the same origin.

### Multi-regression analysis

In this study, we employed electronic nose and GC-MS to detect the Maca odor and volatiles, respectively, and constructed relationships between characteristic chemicals and the origin of Maca. Correlation analysis indicated that some volatiles and sensors had relationships. Considering the sensitivity of electronic nose to some category of chemicals^32^, the volatiles detected by GC-MS were classified into aromatics, esters, aldehydes, olefins, ketones, ethers, alcohols, heterocyclics and sulfides based on the functional groups^33^. Multi-regression equations were then constructed with the response values of electronic nose as dependent variable and different chemicals as independent variables. As shown in Table 3, all sensors had significant relationships with specific Maca volatiles, such as esters, aldehydes, ethers, alcohols, heterocyclics or sulfides. It suggested that the electronic nose coupled with GC-MS is applicable and accurate to predict the origin of Maca. To meet the market requirements and produce better functional Maca, further studies will be conducted to undermine the biosynthesis pathways of Maca volatiles and to identify the hub metabolites for metabolic engineering of Maca.

**Table 3:** Multi-regression analysis of the odor fingerprints and different Maca volatiles.

Sensor	Equation	R^2^	p value
LY2/AA	Y = 0.001X_2_ − 0.154X_6_ + 0.01X_8_ + 0.056X_9_	0.586	0.000
LY2/G	Y = 0.001X_2_ − 0.125X_6_ + 0.007X_8_ + 0.045X_9_	0.587	0.000
LY2/gCT	Y = 0.002X_2_ − 0.311X_6_ − 0.006X_7_ + 0.010X_8_ + 0.089X_9_	0.673	0.000
LY2/gCTL	Y = 0.002X_2_ − 0.363X_6_-0.006X_7_ + 0.019X_8_ + 0.117X_9_	0.597	0.000
LY2/LG	Y = −0.095X_6_ + 0.003X_8_ + 0.030X_9_	0.504	0.000
P10/1	Y = 0.005X_2_ + 0.004X_3_ − 0.935X_6_-0.024X_7_ + 0.025X_8_ + 0.260X_9_	0.718	0.000
P10/2	Y = 0.005X_2_ + 0.004X_3_ − 0.923X_6_ − 0.022X_7_ + 0.031X_8_ + 0.257X_9_	0.709	0.000
P30/1	Y = 0.264 + 0.004X_2_ + 0.003X_3_ − 0.807X_6_ − 0.021X_7_ + 0.023X_8_ + 0.220X_9_	0.681	0.000
P30/2	Y = 0.007X2 + 0.004X3 − 1.170X6 − 0.025X7 + 0.031X8 + 0.310X9	0.695	0.000
P40/1	Y = 0.005X_2_ + 0.004X_3_ − 0.946X_6_ − 0.023X_7_ + 0.029X_8_ + 0.257X_9_	0.714	0.000
P40/2	Y = 0.006X_2_ + 0.004X_3_ − 1.042X_6_ − 0.023X_7_ + 0.032X_8_ + 0.274X_9_	0.716	0.000
PA/2	Y = 0.198 + 0.004X_2_ + 0.004X_3_ − 0.906X_6_ − 0.025X_7_ + 0.027X_8_ + 0.247X_9_	0.695	0.000
T30/1	Y = 0.005X_2_ − 0.952X_6_ − 0.023X_7_ + 0.027X_8_ + 0.253X_9_	0.716	0.000
T40/1	Y = 0.003X_2_ + 0.001X_3_ − 0.420X_6_ − 0.008X_7_ + 0.114X_9_	0.662	0.000
T40/2	Y = 0.004X_2_ + 0.002X_3_ − 0.646X_6_ − 0.014X_7_ + 0.019X_8_ + 0.177X_9_	0.702	0.000
T70/2	Y = 0.005X_2_ + 0.003X_3_ − 0.877X_6_-0.020X_7_ + 0.025X_8_ + 0.228X_9_	0.712	0.000
TA/2	Y = 0.004X_2_ + 0.002X_3_ − 0.642X_6_ − 0.014X_7_ + 0.016X_8_ + 0.176X_9_	0.687	0.000

X_1_, aromatics; X_2_, esters; X_3_, aldehydes; X_4_, olefins; X_5_, ketones; X_6_, ethers; X_7_, alcohols; X_8_, heterocyclics; and X_9_, sulfides.

## Conclusions

Electronic nose coupled with GC-MS was used to identify the origin of Maca from macroscopic and microscopic levels: electronic nose revealed that the odor fingerprint of each Maca sample was distinctively different and specific, while GC-MS confirmed the roles of several characteristic volatiles. Further correlation and multi-regression analyses indicated the close relationship between odor types and components and the roles of each specific volatile. This study proposed a simple, reliable, and efficient method to identify the Maca origin, and revealed the relationship between Maca odors and volatile chemicals. It is of great importance in both scientific and industrial fields.

## Methods

### Maca sampling and powder preparation

Fresh Maca fruits were collected from 101 villages, 18 towns, six cities and two provinces of the main producing area in China in April 6−27, 2016. Approximately 1 kg of each variety (black, purple, and yellow) was sampled. The sampling information is listed in Table S1.

Eight to ten Maca fruits of each sample were washed by tap water, and the fibrous roots and top were removed. After three washes with deionized water, the fruits were sliced to 2-mm-thick pieces and dried at 42 °C in a Yiheng oven (Shanghai, China) to the water content of 6−9%. The Maca slices were then ground into powders, sieved through 60 mesh, and stored at −20 °C before use.

### Detection and analysis of Maca odor

Maca powders (1.5 g) of each sample were placed in a 20-mL headspace bottle, and each sample had triplicate. The odor was detected by an electronic nose (FOX 4000, Alpha MOS, Toulouse, France) coupled with an automatic sampler (HS-100) and a high purity air generator (AG 2301). The electronic nose equipped with 17 detectors (Table S3) is sensitive to strong oxidizing gases, toxic gases, combustible gases, flammable gases, aromatic compounds, and organic compounds. The parameters were set to meet standards that the maximum response values are within 0.25–0.85 and the detectors with maximum values more than 0.95 and minimum values less than 0.05 are as few as possible. To verify the optimized parameters, one Maca sample of each color variety was selected for odor analysis. And the instrument stability was tested by randomly selected 30 Maca samples of six cities. By using the optimized electronic nose parameters, odor characteristics of all Maca samples were detected by 17 sensors (Table S3), and used to construct the BP neuron network. The multi-layer feed-forward algorithm (Fig. S2) was used to identify and classify the Maca origin according to the odor fingerprints detected by electronic nose. The BP neural network was constructed by using the pattern recognition toolbox of MATLAB R2015a.

### GC-MS analysis of the volatiles

Maca powders (1.0 g) of each sample were placed in a 20-mL headspace bottle, and sealed immediately with a silicon lid. After equilibrium in an heated oscillator at 65 °C for 10 min, the volatiles were extracted at 350 rpm for 30 min and placed at the GC injection port for 2 min for desorption. The mass spectrometer (GCMS-TQ8040, Shimadzu, Tokyo, Japan) was equipped with an autosampler AOC-6000 and a InertCapPure-Wax column (30 m × 0.25 mm × 0.25 µm). Solid phase extraction was conducted with the fibers 85 μm PA, 95 μm CAR WR/PDMS, and 75 μm CAR/PDMS (Supelco). Helium (>99.99%) was used as the carrier gas at a constant flow rate of 1 mL/min. The injection volume was 1 mL in splitless mode. The initial oven temperature was held at 50 °C for 5 min, ramped to 250 °C at a rate of 10 °C/ min, and held at 250 °C for 10 min. The temperatures of injector, pressure, split ratio, and electron impact ion source were set to 250 °C, 83.5 kPa, 5:1, and 200 °C, respectively. The qualitative and semi-quantitative data was collected in a full scan mode (m/z 45–450) and multiple reaction monitoring (MRM), respectively.

### Qualitative analysis of Maca volatiles

The full scan data was used for the multivariate analysis of Maca volatiles of different origins. Chemicals were identified by searching against the NIST14 and NIST14S mass spectral libraries, with the similarity of >80% and identities of three chemicals. Using the DPS software, one-way ANOVA analysis was conducted to detect significant (*p* < 0.05) and very significant (*p* < 0.01) differences among the chemicals of Maca from different locations.

### Semi-quantitative analysis of Maca volatiles

MRM scanning coupled with off-flavor analyzer system was used to quantify the chemicals. The off-flavor analyzer system containing the retention time data of approximately 150 chemicals on three chromatographic columns provided the information of odor type and threshold, with *N*-alkanes as an internal reference. Smart MRM was used to create analysis files. To study the Maca clustering of different origin, the normalized data were imported to SIMCA 13.0 (Umetrics) and subjected to orthogonal partial least squares-discriminant analysis (OPLS-DA), and the most different chemicals were identified using the variable importance (VIP) method. In this study, the OPLS-DA plot was constructed with the 85 compounds as variant X and the municipal Maca origins (Donghui, Dali, Lijiang, and Shangri-La) as variant Y. The model quality was described by the values of R^2^X, R^2^Y, and Q^2^. R^2^X and R^2^Y, the proportion of variance, indicated the goodness of fit. Q^2^, the accuracy rate, was calculated by the cross-validation procedure and functioned as an indicator of the predictability of current model. To avoid model over-fitting, a default 7-round cross-validation in SIMCA was performed.

### Correlation and regression analysis

SPSS 22.03 was used to analyze the correlation of Maca volatiles and characteristic odors, in which significant and very significant differences were defined as *p* < 0.05 and *p* < 0.01. Cytoscape 3.6.0 was used to visualize the correlated relationship. And a regression model was constructed with the goal of predicting the Maca origin.

## Supplementary information

Supplementary materials
