# Supplementary material for: Origin identification of Chinese Maca using electronic nose coupled with GC-MS
Source: Sci Rep. 2019 Aug 21;9:12216. doi: 10.1038/s41598-019-47571-0 (PMC6704143; doi:10.1038/s41598-019-47571-0)
Supplement: Supplementary file 1 — Supplementary materials [file 41598_2019_47571_MOESM1_ESM.doc]

**Supplementary materials to**

**Origin identification of Chinese Maca using electronic nose coupled with GC-MS**

Aimin Li**^a,#^**, Shenglin Duan**^b,#^**, Yanting Dang**^b^**, Xi Zhang**^c^**, Kai Xia**^b^**, Shiwei Liu**^b^**, Xiaofeng Han**^b^**, Jian Wen**^b^**, Zijie Li**^a^**, Xi Wang**^b^**, Jia Liu**^b^**, Peng Yuan**^b,*^**, Xiao-Dong Gao**^a,*^**

**^a^** Key Laboratory of Carbohydrate Chemistry and Biotechnology, Ministry of Education, School of Biotechnology, Jiangnan University, Wuxi 214122, China

**^b^** Beijing Key Laboratory of the Innovative Development of Functional Staple and Nutritional Intervention for Chronic Diseases, China National Research Institute of Food and Fermentation Industries Co., LTD., Beijing 100015, China

**^c^** Analytical Instruments Dept. Analytical Application Center Shimadzu Co., LTD, Beijing 100020, China

^#^These authors contributed equally to this work.

* Corresponding authors. Key Laboratory of Carbohydrate Chemistry and Biotechnology, Ministry of Education, School of Biotechnology, Jiangnan University, Wuxi 214122, China; Beijing Key Laboratory of the Innovative Development of Functional Staple and Nutritional Intervention for Chronic Diseases, China National Research Institute of Food and Fermentation Industries Co., LTD., No. 24 Jiuxianqiao Middle Road, Beijing 100015, China. Tel. & fax: +86 510 85197071.

E-mail addresses: [xdgao@jiangnan.edu.cn](mailto:xdgao@jiangnan.edu.cn) (XG); [ypt86@163.com](mailto:ypt86@163.com) (YP)

**Table S1** The collection and geological information of Maca samples.^a^

| No. | Name | Sampling date | Province | City | County | Altitude (m) | Longitude | Latitude |
| --- | --- | --- | --- | --- | --- | --- | --- | --- |
| 1 | YCQ0-1B | April 6, 2016 | Yunnan | Chuxiong | Donghua | 1797 | 101°57.3521′ | 27°98.0059′ |
| 2 | YCQ0-2P | April 6, 2016 | Yunnan | Chuxiong | Donghua | 1797 | 101°57.4681′ | 27°98.4655′ |
| 3 | YCQ0-3Y | April 6, 2016 | Yunnan | Chuxiong | Donghua | 1797 | 101°57.6942′ | 27°99.0716′ |
| 4 | YCDS-1B | April 6, 2016 | Yunnan | Chuxiong | Donghua | 1891 | 101°27.0178′ | 24°57.7305′ |
| 5 | YCDS-2HB | April 6, 2016 | Yunnan | Chuxiong | Donghua | 1889 | 101°27.0549′ | 24°57.7392′ |
| 6 | YDESD-1HY | April 7, 2016 | Yunnan | Dali | Eryuan | 3174 | 100°05.6487′ | 26°10.0169′ |
| 7 | YDESD-2HY | April 7, 2016 | Yunnan | Dali | Eryuan | 3170 | 100°06.0816′ | 26°09.5985′ |
| 8 | YDESD-3B | April 7, 2016 | Yunnan | Dali | Eryuan | 3007 | 100°06.6628′ | 26°08.6832′ |
| 9 | YDESD-4Y | April 7, 2016 | Yunnan | Dali | Eryuan | 3007 | 100°06.6628′ | 26°08.6832′ |
| 10 | YDESD-5P | April 7, 2016 | Yunnan | Dali | Eryuan | 3007 | 100°06.6628′ | 26°08.6832′ |
| 11 | YDHQ-1B | April 7, 2016 | Yunnan | Dali | Heqing | 2276 | 100°09.6807′ | 26°11.5991′ |
| 12 | YDHX-1B | April 9, 2016 | Yunnan | Dali | Heqing | 2723 | 100°07.4657′ | 26°29.0990′ |
| 13 | YDHX-2P | April 9, 2016 | Yunnan | Dali | Heqing | 2723 | 100°07.4657′ | 26°29.0990′ |
| 14 | YDHX-3Y | April 9, 2016 | Yunnan | Dali | Heqing | 2723 | 100°07.4657′ | 26°29.0990′ |
| 15 | YDHX-4B | April 9, 2016 | Yunnan | Dali | Heqing | 2800 | 100°07.2086′ | 26°29.3033′ |
| 16 | YDHX-5P | April 9, 2016 | Yunnan | Dali | Heqing | 2800 | 100°07.2086′ | 26°29.3033′ |
| 17 | YDHX-6Y | April 9, 2016 | Yunnan | Dali | Heqing | 2800 | 100°07.2086′ | 26°29.3033′ |
| 18 | YDHCM-1B | April 9, 2016 | Yunnan | Dali | Heqing | 3000 | 100°04.8782′ | 26°28.1821′ |
| 19 | YDHCM-2P | April 9, 2016 | Yunnan | Dali | Heqing | 3000 | 100°04.8782′ | 26°28.1821′ |
| 20 | YDHCM-3Y | April 9, 2016 | Yunnan | Dali | Heqing | 3000 | 100°04.8782′ | 26°28.1821′ |
| 21 | YDHCH-1B | April 9, 2016 | Yunnan | Dali | Heqing | 3096 | 100°04.7741′ | 26°27.9654′ |
| 22 | YDHCH-2P | April 9, 2016 | Yunnan | Dali | Heqing | 3096 | 100°04.7741′ | 26°27.9654′ |
| 23 | YDHCH-3Y | April 9, 2016 | Yunnan | Dali | Heqing | 3096 | 100°04.7741′ | 26°27.9654′ |
| 24 | YDHCH-4P | April 9, 2016 | Yunnan | Dali | Heqing | 3096 | 100°04.7741′ | 26°27.9654′ |
| 25 | YDHCH-5Y | April 9, 2016 | Yunnan | Dali | Heqing | 3096 | 100°04.7741′ | 26°27.9654′ |
| 26 | YDHCZ-1P | April 9, 2016 | Yunnan | Dali | Heqing | 3080 | 100°03.3910′ | 26°28.3622′ |
| 27 | YDHCZ-2Y | April 9, 2016 | Yunnan | Dali | Heqing | 3080 | 100°03.3910′ | 26°28.3622′ |
| 28 | YDHCB-1B | April 9, 2016 | Yunnan | Dali | Heqing | 3115 | 100°03.6682′ | 26°28.2076′ |
| 29 | YDHCB-2Y | April 9, 2016 | Yunnan | Dali | Heqing | 3115 | 100°03.6682′ | 26°28.2076′ |
| 30 | YDHCC-1B | April 9, 2016 | Yunnan | Dali | Heqing | 3136 | 100°03.2234′ | 26°28.6913′ |
| 31 | YDHCC-2Y | April 9, 2016 | Yunnan | Dali | Heqing | 3136 | 100°03.2234′ | 26°28.6913′ |
| 32 | YDHCC-3P | April 9, 2016 | Yunnan | Dali | Heqing | 3136 | 100°03.2234′ | 26°28.6913′ |
| 33 | YDHCC-4B | April 9, 2016 | Yunnan | Dali | Heqing | 3003 | 100°03.7656′ | 26°30.0632′ |
| 34 | YDHCC-5P | April 9, 2016 | Yunnan | Dali | Heqing | 3003 | 100°03.7656′ | 26°30.0632′ |
| 35 | YDHCC-6Y | April 9, 2016 | Yunnan | Dali | Heqing | 3003 | 100°03.7656′ | 26°30.0632′ |
| 36 | YDJJB-1B | April 9, 2016 | Yunnan | Dali | Jianchuan | 3100 | 100°00.7573′ | 26°31.9962′ |
| 37 | YDJJB-2P | April 9, 2016 | Yunnan | Dali | Jianchuan | 3100 | 100°00.7573′ | 26°31.9962′ |
| 38 | YDJJB-3Y | April 9, 2016 | Yunnan | Dali | Jianchuan | 3100 | 100°00.7573′ | 26°31.9962′ |
| 39 | YKDD-1P | April 10, 2016 | Yunnan | Kunming | Dongchuan | 3000 | 100°12.3922′ | 27°27.4081′ |
| 40 | YLYBZ-1B | April 10, 2016 | Yunnan | Lijiang | Yulong | 3000 | 100°12.3922′ | 27°27.4081′ |
| 41 | YLYBZ-2Y | April 10, 2016 | Yunnan | Lijiang | Yulong | 3000 | 100°12.3922′ | 27°27.4081′ |
| 42 | YLYBZ-3P | April 10, 2016 | Yunnan | Lijiang | Yulong | 3000 | 100°12.3922′ | 27°27.4081′ |
| 43 | YLYJJ-1B | April 10, 2016 | Yunnan | Lijiang | Yulong | 2400 | 99°57.7109′ | 26°39.0641′ |
| 44 | YLYJJ-2P | April 10, 2016 | Yunnan | Lijiang | Yulong | 2400 | 99°57.7109′ | 26°39.0641′ |
| 45 | YLYJJ-3Y | April 10, 2016 | Yunnan | Lijiang | Yulong | 2400 | 99°57.7109′ | 26°39.0641′ |
| 46 | YLYM-1B | April 11, 2016 | Yunnan | Lijiang | Yulong | 4000 | 100°58.3630′ | 27°11.6130′ |
| 47 | YLYM-2P | April 11, 2016 | Yunnan | Lijiang | Yulong | 4000 | 100°58.3630′ | 27°11.6130′ |
| 48 | YLYM-3Y | April 11, 2016 | Yunnan | Lijiang | Yulong | 4000 | 100°58.3630′ | 27°11.6130′ |
| 49 | YLYT-1B | April 11, 2016 | Yunnan | Lijiang | Yulong | 2778 | 100°05.1008′ | 26°47.1337′ |
| 50 | YLYT-2P | April 11, 2016 | Yunnan | Lijiang | Yulong | 2778 | 100°05.1008′ | 26°47.1337′ |
| 51 | YLYT-3Y | April 11, 2016 | Yunnan | Lijiang | Yulong | 2778 | 100°05.1008′ | 26°47.1337′ |
| 52 | YLYH-1B | April 11, 2016 | Yunnan | Lijiang | Yulong | 2875 | 100°04.9092′ | 26°46.7169′ |
| 53 | YLYH-2P | April 11, 2016 | Yunnan | Lijiang | Yulong | 2875 | 100°04.9092′ | 26°46.7169′ |
| 54 | YLYH-3Y | April 11, 2016 | Yunnan | Lijiang | Yulong | 2875 | 100°04.9092′ | 26°46.7169′ |
| 55 | YLYT-4B | April 11, 2016 | Yunnan | Lijiang | Yulong | 2782 | 100°04.8995′ | 26°46.7757′ |
| 56 | YLYT-5P | April 11, 2016 | Yunnan | Lijiang | Yulong | 2782 | 100°04.8995′ | 26°46.7757′ |
| 57 | YLYT-6Y | April 11, 2016 | Yunnan | Lijiang | Yulong | 2782 | 100°04.8995′ | 26°46.7757′ |
| 58 | YLYX-1B | April 11, 2016 | Yunnan | Lijiang | Yulong | 2782 | 100°04.2697′ | 26°46.3289′ |
| 59 | YLYX-2P | April 11, 2016 | Yunnan | Lijiang | Yulong | 2782 | 100°04.2697′ | 26°46.3289′ |
| 60 | YLYX-3Y | April 11, 2016 | Yunnan | Lijiang | Yulong | 2782 | 100°04.2697′ | 26°46.3289′ |
| 61 | YLYJ-1B | April 11, 2016 | Yunnan | Lijiang | Yulong | 2893 | 100°04.0854′ | 26°45.3267′ |
| 62 | YLYJ-2P | April 11, 2016 | Yunnan | Lijiang | Yulong | 2893 | 100°04.0854′ | 26°45.3267′ |
| 63 | YLYJ-3Y | April 11, 2016 | Yunnan | Lijiang | Yulong | 2893 | 100°04.0854′ | 26°45.3267′ |
| 64 | YLYW-1B | April 11, 2016 | Yunnan | Lijiang | Yulong | 2868 | 100°01.3943′ | 26°46.8583′ |
| 65 | YLYW-2P | April 11, 2016 | Yunnan | Lijiang | Yulong | 2868 | 100°01.3943′ | 26°46.8583′ |
| 66 | YLYW-3Y | April 11, 2016 | Yunnan | Lijiang | Yulong | 2868 | 100°01.3943′ | 26°46.8583′ |
| 67 | YLYG-1B | April 11, 2016 | Yunnan | Lijiang | Yulong | 3011 | 100°04.1136′ | 26°43.8333′ |
| 68 | YLYG-2P | April 11, 2016 | Yunnan | Lijiang | Yulong | 3011 | 100°04.1136′ | 26°43.8333′ |
| 69 | YLYG-3Y | April 11, 2016 | Yunnan | Lijiang | Yulong | 3011 | 100°04.1136′ | 26°43.8333′ |
| 70 | YLYQ-1B | April 11, 2016 | Yunnan | Lijiang | Yulong | 2875 | 100°04.6895′ | 26°43.4745′ |
| 71 | YLYQ-2P | April 11, 2016 | Yunnan | Lijiang | Yulong | 2875 | 100°04.6895′ | 26°43.4745′ |
| 72 | YLYQ-3Y | April 11, 2016 | Yunnan | Lijiang | Yulong | 2875 | 100°04.6895′ | 26°43.4745′ |
| 73 | YLYR-1B | April 11, 2016 | Yunnan | Lijiang | Yulong | 3122 | 100°01.7967′ | 26°43.6483′ |
| 74 | YLYR-2P | April 11, 2016 | Yunnan | Lijiang | Yulong | 3122 | 100°01.7967′ | 26°43.6483′ |
| 75 | YLYR-3Y | April 11, 2016 | Yunnan | Lijiang | Yulong | 3122 | 100°01.7967′ | 26°43.6483′ |
| 76 | YLYA-1B | April 11, 2016 | Yunnan | Lijiang | Yulong | 3193 | 100°02.1414′ | 26°42.3784′ |
| 77 | YLYA-2P | April 11, 2016 | Yunnan | Lijiang | Yulong | 3193 | 100°02.1414′ | 26°42.3784′ |
| 78 | YLYA-3Y | April 11, 2016 | Yunnan | Lijiang | Yulong | 3193 | 100°02.1414′ | 26°42.3784′ |
| 79 | YLYB-1B | April 11, 2016 | Yunnan | Lijiang | Yulong | 2647 | 100°03.6497′ | 26°50.8506′ |
| 80 | YLYB-2P | April 11, 2016 | Yunnan | Lijiang | Yulong | 2647 | 100°03.6498′ | 26°50.8507′ |
| 81 | YLYB-3Y | April 11, 2016 | Yunnan | Lijiang | Yulong | 2647 | 100°03.6499′ | 26°50.8508′ |
| 82 | YLYD-1B | April 12, 2016 | Yunnan | Lijiang | Yulong | 3000 | 99°47.8243′ | 26°49.2952′ |
| 83 | YLYD-2P | April 12, 2016 | Yunnan | Lijiang | Yulong | 3000 | 99°47.8243′ | 26°49.2952′ |
| 84 | YLYD-3Y | April 12, 2016 | Yunnan | Lijiang | Yulong | 3000 | 99°47.8243′ | 26°49.2952′ |
| 85 | YLYSR-1B | April 12, 2016 | Yunnan | Lijiang | Yulong | 3600 | 99°48.4611′ | 26°54.2059′ |
| 86 | YLYSR-2P | April 12, 2016 | Yunnan | Lijiang | Yulong | 3600 | 99°48.4611′ | 26°54.2059′ |
| 87 | YLYSR-3Y | April 12, 2016 | Yunnan | Lijiang | Yulong | 3600 | 99°48.4611′ | 26°54.2059′ |
| 88 | YLYSL-1B | April 12, 2016 | Yunnan | Lijiang | Yulong | 3400 | 99°44.8808′ | 26°53.6310′ |
| 89 | YLYSL-2P | April 12, 2016 | Yunnan | Lijiang | Yulong | 3400 | 99°44.8808′ | 26°53.6310′ |
| 90 | YLYSL-3Y | April 12, 2016 | Yunnan | Lijiang | Yulong | 3400 | 99°44.8808′ | 26°53.6310′ |
| 91 | YLYLD-1B | April 13, 2016 | Yunnan | Lijiang | Yulong | 2506 | 99°29.1808′ | 27°11.9824′ |
| 92 | YLYLD-2P | April 13, 2016 | Yunnan | Lijiang | Yulong | 2506 | 99°29.1808′ | 27°11.9824′ |
| 93 | YLYLD-3Y | April 13, 2016 | Yunnan | Lijiang | Yulong | 2506 | 99°29.1808′ | 27°11.9824′ |
| 94 | YLYLS-1B | April 13, 2016 | Yunnan | Lijiang | Yulong | 2800 | 99°28.7173′ | 27°11.8481′ |
| 95 | YLYLS-2P | April 13, 2016 | Yunnan | Lijiang | Yulong | 2800 | 99°28.7173′ | 27°11.8481′ |
| 96 | YLYLS-3Y | April 13, 2016 | Yunnan | Lijiang | Yulong | 2800 | 99°28.7173′ | 27°11.8481′ |
| 97 | YLYLB-1B | April 13, 2016 | Yunnan | Lijiang | Yulong | 2503 | 99°28.5126′ | 27°12.2079′ |
| 98 | YLYLB-2P | April 13, 2016 | Yunnan | Lijiang | Yulong | 2503 | 99°28.5126′ | 27°12.2079′ |
| 99 | YLYLB-3Y | April 13, 2016 | Yunnan | Lijiang | Yulong | 2503 | 99°28.5126′ | 27°12.2079′ |
| 100 | YLYLT-1B | April 13, 2016 | Yunnan | Lijiang | Yulong | 2800 | 99°27.4457′ | 27°11.2258′ |
| 101 | YLYLT-2P | April 13, 2016 | Yunnan | Lijiang | Yulong | 2800 | 99°27.4457′ | 27°11.2258′ |
| 102 | YLYLT-3Y | April 13, 2016 | Yunnan | Lijiang | Yulong | 2800 | 99°27.4457′ | 27°11.2258′ |
| 103 | YLYLA-1B | April 13, 2016 | Yunnan | Lijiang | Yulong | 2800 | 99°27.7223′ | 27°11.0214′ |
| 104 | YLYLA-2P | April 13, 2016 | Yunnan | Lijiang | Yulong | 2800 | 99°27.7223′ | 27°11.0214′ |
| 105 | YLYLA-3Y | April 13, 2016 | Yunnan | Lijiang | Yulong | 2800 | 99°27.7223′ | 27°11.0214′ |
| 106 | YLYLG-1Y | April 13, 2016 | Yunnan | Lijiang | Yulong | 2954 | 99°29.9876′ | 27°08.2501′ |
| 107 | YLYLG-2P | April 13, 2016 | Yunnan | Lijiang | Yulong | 2954 | 99°29.9876′ | 27°08.2501′ |
| 108 | YLYLG-3B | April 13, 2016 | Yunnan | Lijiang | Yulong | 2954 | 99°29.9876′ | 27°08.2501′ |
| 109 | YLYLL-1B | April 13, 2016 | Yunnan | Lijiang | Yulong | 2906 | 99°28.4434′ | 27°08.6676′ |
| 110 | YLYLL-2P | April 13, 2016 | Yunnan | Lijiang | Yulong | 2906 | 99°28.4434′ | 27°08.6676′ |
| 111 | YLYLL-3Y | April 13, 2016 | Yunnan | Lijiang | Yulong | 2906 | 99°28.4434′ | 27°08.6676′ |
| 112 | YXWTC-1B | April 14, 2016 | Yunnan | Shangri-La | Weixi | 2747 | 99°19.7006′ | 27°21.3654′ |
| 113 | YXWTC-2P | April 14, 2016 | Yunnan | Shangri-La | Weixi | 2747 | 99°19.7006′ | 27°21.3654′ |
| 114 | YXWTC-3Y | April 14, 2016 | Yunnan | Shangri-La | Weixi | 2747 | 99°19.7006′ | 27°21.3654′ |
| 115 | YXWTD-1P | April 14, 2016 | Yunnan | Shangri-La | Weixi | 2800 | 99°19.3012′ | 27°20.3084′ |
| 116 | YXWTD-2Y | April 14, 2016 | Yunnan | Shangri-La | Weixi | 2800 | 99°19.3012′ | 27°20.3084′ |
| 117 | YXWTD-3B | April 14, 2016 | Yunnan | Shangri-La | Weixi | 2800 | 99°19.3012′ | 27°20.3084′ |
| 118 | YXWPM-1B | April 14, 2016 | Yunnan | Shangri-La | Weixi | 2902 | 99°16.5160′ | 27°19.5547′ |
| 119 | YXWPM-2P | April 14, 2016 | Yunnan | Shangri-La | Weixi | 2902 | 99°16.5160′ | 27°19.5547′ |
| 120 | YXWPM-3Y | April 14, 2016 | Yunnan | Shangri-La | Weixi | 2902 | 99°16.5160′ | 27°19.5547′ |
| 121 | YXWBL-1B | April 14, 2016 | Yunnan | Shangri-La | Weixi | 2875 | 99°16.0648′ | 27°17.4307′ |
| 122 | YXWBL-2P | April 14, 2016 | Yunnan | Shangri-La | Weixi | 2875 | 99°16.0648′ | 27°17.4307′ |
| 123 | YXWBL-3Y | April 14, 2016 | Yunnan | Shangri-La | Weixi | 2875 | 99°16.0648′ | 27°17.4307′ |
| 124 | YXWBH-1B | April 14, 2016 | Yunnan | Shangri-La | Weixi | 3020 | 99°17.0731′ | 27°15.8385′ |
| 125 | YXWBH-2P | April 14, 2016 | Yunnan | Shangri-La | Weixi | 3020 | 99°17.0731′ | 27°15.8385′ |
| 126 | YXWBH-3Y | April 14, 2016 | Yunnan | Shangri-La | Weixi | 3020 | 99°17.0731′ | 27°15.8385′ |
| 127 | YXWYT-1B | April 14, 2016 | Yunnan | Shangri-La | Weixi | 2553 | 99°22.3658′ | 27°07.1343′ |
| 128 | YXWYT-2P | April 14, 2016 | Yunnan | Shangri-La | Weixi | 2553 | 99°22.3658′ | 27°07.1343′ |
| 129 | YXWYT-3Y | April 14, 2016 | Yunnan | Shangri-La | Weixi | 2553 | 99°22.3658′ | 27°07.1343′ |
| 130 | YXWL-1B | April 14, 2016 | Yunnan | Shangri-La | Weixi | 2427 | 99°21.8604′ | 27°07.1388′ |
| 131 | YXWL-2P | April 14, 2016 | Yunnan | Shangri-La | Weixi | 2427 | 99°21.8604′ | 27°07.1388′ |
| 132 | YXWL-3Y | April 14, 2016 | Yunnan | Shangri-La | Weixi | 2427 | 99°21.8604′ | 27°07.1388′ |
| 133 | YXWA-1B | April 14, 2016 | Yunnan | Shangri-La | Weixi | 2642 | 99°23.2838′ | 27°06.3964′ |
| 134 | YXWA-2P | April 14, 2016 | Yunnan | Shangri-La | Weixi | 2642 | 99°23.2838′ | 27°06.3964′ |
| 135 | YXWA-3Y | April 14, 2016 | Yunnan | Shangri-La | Weixi | 2642 | 99°23.2838′ | 27°06.3964′ |
| 136 | YXJG-1B | April 15, 2016 | Yunnan | Shangri-La | Weixi | 3292 | 99°38.6690′ | 27°54.2507′ |
| 137 | YXJG-2P | April 15, 2016 | Yunnan | Shangri-La | Weixi | 3292 | 99°38.6690′ | 27°54.2507′ |
| 138 | YXJG-3Y | April 15, 2016 | Yunnan | Shangri-La | Weixi | 3292 | 99°38.6690′ | 27°54.2507′ |
| 139 | YXJY-1B | April 15, 2016 | Yunnan | Shangri-La | Weixi | 3285 | 99°39.7552′ | 27°52.3717′ |
| 140 | YXJY-2P | April 15, 2016 | Yunnan | Shangri-La | Weixi | 3285 | 99°39.7552′ | 27°52.3717′ |
| 141 | YXJY-3Y | April 15, 2016 | Yunnan | Shangri-La | Weixi | 3285 | 99°39.7552′ | 27°52.3717′ |
| 142 | YXJN-1B | April 16, 2016 | Yunnan | Shangri-La | Weixi | 3295 | 99°37.3490′ | 27°52.6147′ |
| 143 | YXJN-2P | April 16, 2016 | Yunnan | Shangri-La | Weixi | 3295 | 99°37.3490′ | 27°52.6147′ |
| 144 | YXJN-3Y | April 16, 2016 | Yunnan | Shangri-La | Weixi | 3295 | 99°37.3490′ | 27°52.6147′ |
| 145 | YXXB-1B | April 16, 2016 | Yunnan | Shangri-La | Weixi | 3176 | 99°48.6367′ | 27°30.4177′ |
| 146 | YXXB-2P | April 16, 2016 | Yunnan | Shangri-La | Weixi | 3176 | 99°48.6367′ | 27°30.4177′ |
| 147 | YXXB-3Y | April 16, 2016 | Yunnan | Shangri-La | Weixi | 3176 | 99°48.6367′ | 27°30.4177′ |
| 148 | YXXB-4H | April 16, 2016 | Yunnan | Shangri-La | Weixi | 3176 | 99°48.6367′ | 27°30.4177′ |
| 149 | YXHH-1P | April 16, 2016 | Yunnan | Shangri-La | Weixi | 3171 | 99°53.2606′ | 27°24.1086′ |
| 150 | YXHH-2Y | April 16, 2016 | Yunnan | Shangri-La | Weixi | 3171 | 99°53.2606′ | 27°24.1086′ |
| 151 | YXHH-3B | April 16, 2016 | Yunnan | Shangri-La | Weixi | 3171 | 99°53.2606′ | 27°24.1086′ |
| 152 | YXHH-4P | April 16, 2016 | Yunnan | Shangri-La | Weixi | 3176 | 99°53.6208′ | 27°24.5177′ |
| 153 | YXHH-5Y | April 16, 2016 | Yunnan | Shangri-La | Weixi | 3176 | 99°53.6208′ | 27°24.5177′ |
| 154 | YXHH-6B | April 16, 2016 | Yunnan | Shangri-La | Weixi | 3176 | 99°53.6208′ | 27°24.5177′ |
| 155 | YXHC-1B | April 16, 2016 | Yunnan | Shangri-La | Weixi | 3280 | 99°52.9330′ | 27°23.8894′ |
| 156 | YXHC-2P | April 16, 2016 | Yunnan | Shangri-La | Weixi | 3280 | 99°52.9330′ | 27°23.8894′ |
| 157 | YXHC-3Y | April 16, 2016 | Yunnan | Shangri-La | Weixi | 3280 | 99°52.9330′ | 27°23.8894′ |
| 158 | YXHW-1B | April 16, 2016 | Yunnan | Shangri-La | Weixi | 3163 | 99°52.8953′ | 27°23.5479′ |
| 159 | YXHW-2P | April 16, 2016 | Yunnan | Shangri-La | Weixi | 3163 | 99°52.8953′ | 27°23.5479′ |
| 160 | YXHW-3Y | April 16, 2016 | Yunnan | Shangri-La | Weixi | 3163 | 99°52.8953′ | 27°23.5479′ |
| 161 | YXJS-1B | April 17, 2016 | Yunnan | Shangri-La | Weixi | 3270 | 99°40.1358′ | 27°47.7222′ |
| 162 | YXJS-2P | April 17, 2016 | Yunnan | Shangri-La | Weixi | 3270 | 99°40.1358′ | 27°47.7222′ |
| 163 | YXJS-3Y | April 17, 2016 | Yunnan | Shangri-La | Weixi | 3270 | 99°40.1358′ | 27°47.7222′ |
| 164 | YXJD-1B | April 17, 2016 | Yunnan | Shangri-La | Weixi | 3305 | 99°41.6321′ | 27°45.0187′ |
| 165 | YXJD-2Y | April 17, 2016 | Yunnan | Shangri-La | Weixi | 3305 | 99°41.6321′ | 27°45.0187′ |
| 166 | YXXT-1B | April 17, 2016 | Yunnan | Shangri-La | Weixi | 3278 | 99°43.7893′ | 27°40.3530′ |
| 167 | YXXT-2P | April 17, 2016 | Yunnan | Shangri-La | Weixi | 3278 | 99°43.7893′ | 27°40.3530′ |
| 168 | YXXT-3Y | April 17, 2016 | Yunnan | Shangri-La | Weixi | 3278 | 99°43.7893′ | 27°40.3530′ |
| 169 | YXXG-1P | April 17, 2016 | Yunnan | Shangri-La | Weixi | 3264 | 99°43.6877′ | 27°40.0221′ |
| 170 | YXXG-2Y | April 17, 2016 | Yunnan | Shangri-La | Weixi | 3264 | 99°43.6877′ | 27°40.0221′ |
| 171 | YXXG-3B | April 17, 2016 | Yunnan | Shangri-La | Weixi | 3264 | 99°43.6877′ | 27°40.0221′ |
| 172 | YXXR-1B | April 17, 2016 | Yunnan | Shangri-La | Weixi | 3285 | 99°42.7014′ | 27°39.0891′ |
| 173 | YXXR-2P | April 17, 2016 | Yunnan | Shangri-La | Weixi | 3285 | 99°42.7014′ | 27°39.0891′ |
| 174 | YXXR-3Y | April 17, 2016 | Yunnan | Shangri-La | Weixi | 3285 | 99°42.7014′ | 27°39.0891′ |
| 175 | YXXN-1B | April 17, 2016 | Yunnan | Shangri-La | Weixi | 3263 | 99°43.3890′ | 27°38.8078′ |
| 176 | YXXN-2P | April 17, 2016 | Yunnan | Shangri-La | Weixi | 3263 | 99°43.3890′ | 27°38.8078′ |
| 177 | YXXN-3Y | April 17, 2016 | Yunnan | Shangri-La | Weixi | 3263 | 99°43.3890′ | 27°38.8078′ |
| 178 | YXXZ-1B | April 17, 2016 | Yunnan | Shangri-La | Weixi | 3250 | 99°44.0237′ | 27°36.7142′ |
| 179 | YXXZ-2P | April 17, 2016 | Yunnan | Shangri-La | Weixi | 3250 | 99°44.0237′ | 27°36.7142′ |
| 180 | YXXZ-3Y | April 17, 2016 | Yunnan | Shangri-La | Weixi | 3250 | 99°44.0237′ | 27°36.7142′ |
| 181 | YXXQ-1B | April 17, 2016 | Yunnan | Shangri-La | Weixi | 3256 | 99°44.0247′ | 27°36.1144′ |
| 182 | YXXQ-2P | April 17, 2016 | Yunnan | Shangri-La | Weixi | 3256 | 99°44.0247′ | 27°36.1144′ |
| 183 | YXXQ-3Y | April 17, 2016 | Yunnan | Shangri-La | Weixi | 3256 | 99°44.0247′ | 27°36.1144′ |
| 184 | YXXK-1B | April 17, 2016 | Yunnan | Shangri-La | Weixi | 3247 | 99°45.0042′ | 27°35.6315′ |
| 185 | YXXK-2P | April 17, 2016 | Yunnan | Shangri-La | Weixi | 3247 | 99°45.0042′ | 27°35.6315′ |
| 186 | YXXK-3Y | April 17, 2016 | Yunnan | Shangri-La | Weixi | 3247 | 99°45.0042′ | 27°35.6315′ |
| 187 | YXXD-1B | April 17, 2016 | Yunnan | Shangri-La | Weixi | 3245 | 99°44.5109′ | 27°35.4447′ |
| 188 | YXXD-2P | April 17, 2016 | Yunnan | Shangri-La | Weixi | 3245 | 99°44.5109′ | 27°35.4447′ |
| 189 | YXXD-3Y | April 17, 2016 | Yunnan | Shangri-La | Weixi | 3245 | 99°44.5109′ | 27°35.4447′ |
| 190 | YXXD-1B | April 17, 2016 | Yunnan | Shangri-La | Weixi | 3248 | 99°45.2908′ | 27°34.7244′ |
| 191 | YXXD-2P | April 17, 2016 | Yunnan | Shangri-La | Weixi | 3248 | 99°45.2908′ | 27°34.7244′ |
| 192 | YXXX-1B | April 18, 2016 | Yunnan | Shangri-La | Weixi | 3182 | 99°48.0925′ | 27°31.3935′ |
| 193 | YXXX-2P | April 18, 2016 | Yunnan | Shangri-La | Weixi | 3182 | 99°48.0925′ | 27°31.3935′ |
| 194 | YXXX-3Y | April 18, 2016 | Yunnan | Shangri-La | Weixi | 3182 | 99°48.0925′ | 27°31.3935′ |
| 195 | YXXS-1B | April 18, 2016 | Yunnan | Shangri-La | Weixi | 3114 | 99°48.4975′ | 27°30.1452′ |
| 196 | YXXS-2P | April 18, 2016 | Yunnan | Shangri-La | Weixi | 3114 | 99°48.4975′ | 27°30.1452′ |
| 197 | YXXS-3Y | April 18, 2016 | Yunnan | Shangri-La | Weixi | 3114 | 99°48.4975′ | 27°30.1452′ |
| 198 | YXHD-1B | April 18, 2016 | Yunnan | Shangri-La | Weixi | 3195 | 99°54.3017′ | 27°22.2262′ |
| 199 | YXHD-2P | April 18, 2016 | Yunnan | Shangri-La | Weixi | 3195 | 99°54.3017′ | 27°22.2262′ |
| 200 | YXHD-3Y | April 18, 2016 | Yunnan | Shangri-La | Weixi | 3195 | 99°54.3017′ | 27°22.2262′ |
| 201 | YXHE-1B | April 18, 2016 | Yunnan | Shangri-La | Weixi | 2923 | 99°54.6413′ | 27°21.4916′ |
| 202 | YXHE-2P | April 18, 2016 | Yunnan | Shangri-La | Weixi | 2923 | 99°54.6413′ | 27°21.4916′ |
| 203 | YXHE-3Y | April 18, 2016 | Yunnan | Shangri-La | Weixi | 2923 | 99°54.6413′ | 27°21.4916′ |
| 204 | YLYAG-1B | April 19, 2016 | Yunnan | Lijiang | Yulong | 3600 | 100°21,.6381′ | 27°26.0960′ |
| 205 | YLYAG-2P | April 19, 2016 | Yunnan | Lijiang | Yulong | 3600 | 100°21,.6381′ | 27°26.0960′ |
| 206 | YLYAG-3Y | April 19, 2016 | Yunnan | Lijiang | Yulong | 3600 | 100°21,.6381′ | 27°26.0960′ |
| 207 | YLYAH-1B | April 19, 2016 | Yunnan | Lijiang | Yulong | 3219 | 100°17.6567′ | 27°28.5907′ |
| 208 | YLYAH-2P | April 19, 2016 | Yunnan | Lijiang | Yulong | 3219 | 100°17.6567′ | 27°28.5907′ |
| 209 | YLYAH-3Y | April 19, 2016 | Yunnan | Lijiang | Yulong | 3219 | 100°17.6567′ | 27°28.5907′ |
| 210 | YLYFB-1B | April 19, 2016 | Yunnan | Lijiang | Yulong | 2783 | 100°21.2131′ | 27°38.2427′ |
| 211 | YLYFB-2P | April 19, 2016 | Yunnan | Lijiang | Yulong | 2783 | 100°21.2131′ | 27°38.2427′ |
| 212 | YLYFB-3Y | April 19, 2016 | Yunnan | Lijiang | Yulong | 2783 | 100°21.2131′ | 27°38.2427′ |
| 213 | YLNYL -1B | April 20, 2016 | Yunnan | Lijiang | Ninglang | 2783 | 100°37.6611′ | 27°44.6854′ |
| 214 | YLNYL -2P | April 20, 2016 | Yunnan | Lijiang | Ninglang | 2783 | 100°37.6611′ | 27°44.6854′ |
| 215 | YLNYL -3Y | April 20, 2016 | Yunnan | Lijiang | Ninglang | 2783 | 100°37.6611′ | 27°44.6854′ |
| 216 | YLNYB -1B | April 20, 2016 | Yunnan | Lijiang | Ninglang | 2686 | 100°41.0546′ | 27°49.8504′ |
| 217 | YLNYB-2P | April 20, 2016 | Yunnan | Lijiang | Ninglang | 2686 | 100°41.0546′ | 27°49.8504′ |
| 218 | YLNYB-3Y | April 20, 2016 | Yunnan | Lijiang | Ninglang | 2686 | 100°41.0546′ | 27°49.8504′ |
| 219 | YLNYA-1B | April 20, 2016 | Yunnan | Lijiang | Ninglang | 4300 | 100°41.3565′ | 27°50.8638′ |
| 220 | YLNYA-2P | April 20, 2016 | Yunnan | Lijiang | Ninglang | 4300 | 100°41.3565′ | 27°50.8638′ |
| 221 | YLNYA-3Y | April 20, 2016 | Yunnan | Lijiang | Ninglang | 4300 | 100°41.3565′ | 27°50.8638′ |
| 222 | YLNYC -1B | April 20, 2016 | Yunnan | Lijiang | Ninglang | 4200 | 100°41.3360′ | 27°50.8693′ |
| 223 | YLNYC-2P | April 20, 2016 | Yunnan | Lijiang | Ninglang | 4200 | 100°41.3360′ | 27°50.8693′ |
| 224 | YLNYC-3Y | April 20, 2016 | Yunnan | Lijiang | Ninglang | 4200 | 100°41.3360′ | 27°50.8693′ |
| 225 | YLNYS-1B | April 20, 2016 | Yunnan | Lijiang | Ninglang | 2960 | 100°40.8868′ | 27°46.1707′ |
| 226 | YLNYS-2P | April 20, 2016 | Yunnan | Lijiang | Ninglang | 2960 | 100°40.8868′ | 27°46.1707′ |
| 227 | YLNYS-3Y | April 20, 2016 | Yunnan | Lijiang | Ninglang | 2960 | 100°40.8868′ | 27°46.1707′ |
| 228 | YLNYM-1B | April 20, 2016 | Yunnan | Lijiang | Ninglang | 3300 | 100°40.0990′ | 27°44.7647′ |
| 229 | YLNYM-2P | April 21, 2016 | Yunnan | Lijiang | Ninglang | 3300 | 100°40.0990′ | 27°44.7647′ |
| 230 | YLNYM-3Y | April 21, 2016 | Yunnan | Lijiang | Ninglang | 3300 | 100°40.0990′ | 27°44.7647′ |
| 231 | YLNXD-1B | April 21, 2016 | Yunnan | Lijiang | Ninglang | 2846 | 100°54.8095′ | 27°11.0849′ |
| 232 | YLNXD-2P | April 21, 2016 | Yunnan | Lijiang | Ninglang | 2846 | 100°54.8095′ | 27°11.0849′ |
| 233 | YLNXD-3Y | April 21, 2016 | Yunnan | Lijiang | Ninglang | 2846 | 100°54.8095′ | 27°11.0849′ |
| 234 | YLNXP-1B | April 21, 2016 | Yunnan | Lijiang | Ninglang | 3800 | 100°55.2949′ | 27°11.2022′ |
| 235 | YLNXP-2P | April 21, 2016 | Yunnan | Lijiang | Ninglang | 3800 | 100°55.2949′ | 27°11.2022′ |
| 236 | YLNXP-3Y | April 21, 2016 | Yunnan | Lijiang | Ninglang | 3800 | 100°55.2949′ | 27°11.2022′ |
| 237 | YLNLM-1B | April 21, 2016 | Yunnan | Lijiang | Ninglang | 3302 | 100°57.5132′ | 27°11.7489′ |
| 238 | YLNLM-2P | April 21, 2016 | Yunnan | Lijiang | Ninglang | 3302 | 100°57.5132′ | 27°11.7489′ |
| 239 | YLNLM-3Y | April 21, 2016 | Yunnan | Lijiang | Ninglang | 3302 | 100°57.5132′ | 27°11.7489′ |
| 240 | YLNLH-1B | April 21, 2016 | Yunnan | Lijiang | Ninglang | 3293 | 100°59.0953′ | 27°11.1737′ |
| 241 | YLNLH-2P | April 21, 2016 | Yunnan | Lijiang | Ninglang | 3293 | 100°59.0953′ | 27°11.1737′ |
| 242 | YLNLH-3Y | April 21, 2016 | Yunnan | Lijiang | Ninglang | 3293 | 100°59.0953′ | 27°11.1737′ |
| 243 | YLNLJ-1B | April 21, 2016 | Yunnan | Lijiang | Ninglang | 3248 | 101°00.4108′ | 27°10.6288′ |
| 244 | YLNLJ-2P | April 21, 2016 | Yunnan | Lijiang | Ninglang | 3248 | 101°00.4108′ | 27°10.6288′ |
| 245 | YLNLJ-3Y | April 21, 2016 | Yunnan | Lijiang | Ninglang | 3248 | 101°00.4108′ | 27°10.6288′ |
| 246 | YLNPP-1B | April 21, 2016 | Yunnan | Lijiang | Ninglang | 3200 | 101°00.3181′ | 27°04.0119′ |
| 247 | YLNPP-2P | April 21, 2016 | Yunnan | Lijiang | Ninglang | 3200 | 101°00.3181′ | 27°04.0119′ |
| 248 | YLNPP-3Y | April 21, 2016 | Yunnan | Lijiang | Ninglang | 3200 | 101°00.3181′ | 27°04.0119′ |
| 249 | YLSRH-1B | April 22, 2016 | Yunnan | Lijiang | Yongsheng | 2800 | 101°05.3912′ | 26°27.3195′ |
| 250 | YLSRH-2P | April 22, 2016 | Yunnan | Lijiang | Yongsheng | 2800 | 101°05.3912′ | 26°27.3195′ |
| 251 | SXHLL-1B | April 23, 2016 | Sichuan | Xichang | Huidong | 2900 | 102°42.8617′ | 26°31.5032′ |
| 252 | SXHLL -2P | April 23, 2016 | Sichuan | Xichang | Huidong | 2900 | 102°42.8617′ | 26°31.5032′ |
| 253 | SXHLL-3Y | April 23, 2016 | Sichuan | Xichang | Huidong | 2900 | 102°42.8617′ | 26°31.5032′ |
| 254 | SXHLL-4B | April 23, 2016 | Sichuan | Xichang | Huidong | 2900 | 102°42.8617′ | 26°31.5032′ |
| 255 | SXHLL-5P | April 23, 2016 | Sichuan | Xichang | Huidong | 2900 | 102°42.8617′ | 26°31.5032′ |
| 256 | SXHLL-6Y | April 23, 2016 | Sichuan | Xichang | Huidong | 2900 | 102°42.8617′ | 26°31.5032′ |
| 257 | SXHBA-1B | April 24, 2016 | Sichuan | Xichang | Huidong | 3000 | 102°47.0108′ | 26°33.2279′ |
| 258 | SXHBA -2P | April 24, 2016 | Sichuan | Xichang | Huidong | 3000 | 102°47.0108′ | 26°33.2279′ |
| 259 | SXHBA-3Y | April 24, 2016 | Sichuan | Xichang | Huidong | 3000 | 102°47.0108′ | 26°33.2279′ |
| 260 | SXHMX-1B | April 24, 2016 | Sichuan | Xichang | Huidong | 2772 | 102°48.3604′ | 26°30.8589′ |
| 261 | SXHMX-2P | April 24, 2016 | Sichuan | Xichang | Huidong | 2772 | 102°48.3604′ | 26°30.8589′ |
| 262 | SXHMX-3Y | April 24, 2016 | Sichuan | Xichang | Huidong | 2772 | 102°48.3604′ | 26°30.8589′ |
| 263 | SXHMS-1B | April 24, 2016 | Sichuan | Xichang | Huidong | 2753 | 102°48.0588′ | 26°31.1949′ |
| 264 | SXHMS-2P | April 24, 2016 | Sichuan | Xichang | Huidong | 2753 | 102°48.0588′ | 26°31.1949′ |
| 265 | SXHMS-3Y | April 24, 2016 | Sichuan | Xichang | Huidong | 2753 | 102°48.0588′ | 26°31.1949′ |
| 266 | SXHML-1B | April 24, 2016 | Sichuan | Xichang | Huidong | 2900 | 102°48.6072′ | 26°32.6018′ |
| 267 | SXHML-2P | April 24, 2016 | Sichuan | Xichang | Huidong | 2900 | 102°48.6072′ | 26°32.6018′ |
| 268 | SXHML-3Y | April 24, 2016 | Sichuan | Xichang | Huidong | 2900 | 102°48.6072′ | 26°32.6018′ |
| 269 | SXHMQ-1B | April 24, 2016 | Sichuan | Xichang | Huidong | 2600 | 102°46.8246′ | 26°31.2042′ |
| 270 | SXHMQ-2P | April 24, 2016 | Sichuan | Xichang | Huidong | 2600 | 102°46.8246′ | 26°31.2042′ |
| 271 | SXHMQ-3Y | April 24, 2016 | Sichuan | Xichang | Huidong | 2600 | 102°46.8246′ | 26°31.2042′ |
| 272 | SXHMB-1B | April 24, 2016 | Sichuan | Xichang | Huidong | 2610 | 102°47.0345′ | 26°31.4505′ |
| 273 | SXHMB-2P | April 24, 2016 | Sichuan | Xichang | Huidong | 2610 | 102°47.0345′ | 26°31.4505′ |
| 274 | SXHMB-3Y | April 24, 2016 | Sichuan | Xichang | Huidong | 2610 | 102°47.0345′ | 26°31.4505′ |
| 275 | SXHDD-1B | April 24, 2016 | Sichuan | Xichang | Huidong | 2800 | 101°40.9990′ | 27°38.3889′ |
| 276 | SXHDD-2P | April 24, 2016 | Sichuan | Xichang | Huidong | 2800 | 101°40.9990′ | 27°38.3889′ |
| 277 | SXHWW-1B | April 25, 2016 | Sichuan | Xichang | Huidong | 2800 | 102°42.8228′ | 26°21.2768′ |
| 278 | SXHBB-1B | April 26, 2016 | Sichuan | Xichang | Huidong | 2900 | 102°38.1010′ | 26°30.5564′ |
| 279 | SXHQS-1P | April 26, 2016 | Sichuan | Xichang | Huidong | 2928 | 102°55.7709′ | 26°39.1894′ |
| 280 | SXHQS-2Y | April 26, 2016 | Sichuan | Xichang | Huidong | 2928 | 102°55.7709′ | 26°39.1894′ |
| 281 | SXHQS-3B | April 26, 2016 | Sichuan | Xichang | Huidong | 2928 | 102°55.7709′ | 26°39.1894′ |
| 282 | SXHQL -1B | April 26, 2016 | Sichuan | Xichang | Huidong | 2915 | 102°55.9394′ | 26°39.4788′ |
| 283 | SXHQL-2P | April 26, 2016 | Sichuan | Xichang | Huidong | 2915 | 102°55.9394′ | 26°39.4788′ |
| 284 | SXHQL-3Y | April 26, 2016 | Sichuan | Xichang | Huidong | 2915 | 102°55.9394′ | 26°39.4788′ |
| 285 | SXHQA-1B | April 26, 2016 | Sichuan | Xichang | Huidong | 2700 | 102°54.0647′ | 26°39.5720′ |
| 286 | SXHQA-2Y | April 26, 2016 | Sichuan | Xichang | Huidong | 2700 | 102°54.0647′ | 26°39.5720′ |
| 287 | SXHQA-3P | April 26, 2016 | Sichuan | Xichang | Huidong | 2700 | 102°54.0647′ | 26°39.5720′ |
| 288 | SXHLE-1B | April 26, 2016 | Sichuan | Xichang | Huidong | 2760 | 102°43.3001′ | 26°32.6976′ |
| 289 | SXHLE-2P | April 26, 2016 | Sichuan | Xichang | Huidong | 2760 | 102°43.3001′ | 26°32.6976′ |
| 290 | SXHLE-3Y | April 26, 2016 | Sichuan | Xichang | Huidong | 2760 | 102°43.3001′ | 26°32.6976′ |
| 291 | SXHLZ-1H | April 26, 2016 | Sichuan | Xichang | Huidong | 2923 | 102°43.5000′ | 26°32.0397′ |
| 292 | SXHLZ-2B | April 26, 2016 | Sichuan | Xichang | Huidong | 2923 | 102°43.5000′ | 26°32.0397′ |
| 293 | SXHLZ-3P | April 26, 2016 | Sichuan | Xichang | Huidong | 2923 | 102°43.5000′ | 26°32.0397′ |
| 294 | SXHLZ-4Y | April 26, 2016 | Sichuan | Xichang | Huidong | 2923 | 102°43.5000′ | 26°32.0397′ |
| 295 | SXHMA-1B | April 26, 2016 | Sichuan | Xichang | Huidong | 2900 | 101°58.4421′ | 27°19.2387′ |
| 296 | YKLML-1B | April 27, 2016 | Yunnan | Kunming | Luquan | 2802 | 102°34.2609′ | 26°06.1268′ |
| 297 | YKLML-2P | April 27, 2016 | Yunnan | Kunming | Luquan | 2802 | 102°34.2609′ | 26°06.1268′ |
| 298 | YKLML-3Y | April 27, 2016 | Yunnan | Kunming | Luquan | 2802 | 102°34.2609′ | 26°06.1268′ |
| 299 | YKLMC-1P | April 27, 2016 | Yunnan | Kunming | Luquan | 2794 | 102°34.3720′ | 26°07.4335′ |
| 300 | YKLMC-2Y | April 27, 2016 | Yunnan | Kunming | Luquan | 2794 | 102°34.3720′ | 26°07.4335′ |
| 301 | YKLMA-1B | April 27, 2016 | Yunnan | Kunming | Luquan | 2790 | 102°32.5609′ | 26°05.0964′ |
| 302 | YKLMA-2P | April 27, 2016 | Yunnan | Kunming | Luquan | 2790 | 102°32.5609′ | 26°05.0964′ |
| 303 | YKLMA-3Y | April 27, 2016 | Yunnan | Kunming | Luquan | 2790 | 102°32.5609′ | 26°05.0964′ |

^a^ B, black; P, purple; and Y, yellow.

**Table S2** Relative standard deviations of the Maca odors detected by the electronic nose.

| No. | Sample | LY2/LG | LY2/G | LY2/AA | T30/1 | P10/1 | P10/2 | P40/1 | T70/2 | P40/2 | TA/2 |
| --- | --- | --- | --- | --- | --- | --- | --- | --- | --- | --- | --- |
| 1 | SXHBA-2P | 4.996 | −11.215 | −12.494 | 1.114 | 3.004 | 3.056 | 1.907 | 1.596 | 2.491 | 3.982 |
| 2 | SXHLE-3Y | 1.718 | −8.729 | −10.118 | 2.008 | 4.618 | 4.376 | 2.764 | 2.722 | 3.756 | 5.959 |
| 3 | SXHLL-3Y | 4.647 | −11.303 | −12.216 | 1.317 | 3.226 | 3.219 | 2.167 | 1.910 | 2.939 | 4.645 |
| 4 | SXHMB-1B | 5.312 | −10.312 | −11.173 | 0.714 | 1.735 | 1.649 | 0.833 | 1.040 | 1.308 | 2.800 |
| 5 | SXHMB-2P | 5.480 | −10.014 | −11.071 | 0.974 | 2.220 | 2.080 | 1.120 | 1.366 | 1.794 | 3.497 |
| 6 | SXHMB-3Y | 5.023 | −10.864 | −12.000 | 0.948 | 2.177 | 2.033 | 1.070 | 1.375 | 1.759 | 3.391 |
| 7 | SXHQS-3B | 3.771 | −9.232 | −10.125 | 0.982 | 2.565 | 2.619 | 1.562 | 1.395 | 1.984 | 3.270 |
| 8 | YCDS-2HB | 0.523 | −2.408 | −2.651 | 0.622 | 1.501 | 0.966 | 0.502 | 0.863 | 0.739 | 2.965 |
| 9 | YDHCC-4B | 1.733 | −0.7802 | −0.7929 | 0.562 | 1.865 | 0.8142 | 0.342 | 1.020 | 0.732 | 3.193 |
| 10 | YDHCH-3Y | 5.287 | −13.525 | −13.724 | 1.666 | 3.684 | 3.451 | 2.151 | 2.356 | 3.010 | 5.537 |
| 11 | YDHCM-2P | 10.68 | −14.381 | −14.711 | 2.244 | 3.764 | 3.332 | 2.401 | 2.760 | 3.430 | 6.548 |
| 12 | YKLMA-1B | 4.306 | −9.683 | −11.177 | 0.809 | 2.906 | 2.690 | 1.254 | 1.071 | 1.746 | 4.161 |
| 13 | YKLMA-2P | 4.838 | −10.261 | −12.328 | 0.759 | 2.402 | 2.260 | 1.006 | 0.941 | 1.405 | 3.348 |
| 14 | YKLML-1B | 1.425 | −2.032 | −1.808 | 0.422 | 1.046 | 0.6064 | 0.3367 | 0.747 | 0.485 | 2.334 |
| 15 | YLNLJ-2P | 6.103 | −10.639 | −11.477 | 3.541 | 4.662 | 4.667 | 3.775 | 3.950 | 4.315 | 5.874 |
| 16 | YLNLJ-3Y | 8.869 | −12.435 | −13.207 | 4.089 | 5.539 | 5.452 | 4.493 | 4.690 | 5.041 | 7.561 |
| 17 | YLNLM-1B | 5.444 | −8.933 | −10.122 | 2.232 | 4.558 | 4.229 | 2.902 | 3.003 | 3.125 | 5.128 |
| 18 | YLNLM-2P | 4.450 | −12.787 | −12.849 | 2.965 | 5.254 | 5.244 | 3.699 | 3.657 | 4.068 | 6.358 |
| 19 | YLNLM-3Y | 3.661 | −8.281 | −10.017 | 1.734 | 4.349 | 4.173 | 2.743 | 2.465 | 3.055 | 4.596 |
| 20 | YLNPP-1B | 6.969 | −10.519 | −10.719 | 0.912 | 2.426 | 2.294 | 1.417 | 1.137 | 1.991 | 4.284 |
| 21 | YLNPP-3Y | 6.740 | −13.963 | −14.932 | 1.283 | 3.520 | 3.282 | 2.185 | 1.604 | 2.657 | 5.296 |
| 22 | YLNXP-2P | 3.876 | −10.903 | −12.235 | 1.486 | 2.859 | 3.209 | 2.345 | 2.006 | 2.525 | 3.890 |
| 23 | YLNXP-3Y | 5.130 | −11.588 | −12.491 | 1.561 | 2.838 | 3.251 | 1.974 | 1.892 | 2.506 | 3.842 |
| 24 | YLNYL-3Y | 8.337 | −10.689 | −12.442 | 3.241 | 4.855 | 4.803 | 3.952 | 3.985 | 4.015 | 5.616 |
| 25 | YLSRH-2P | 7.746 | −10.228 | −11.612 | 3.215 | 5.133 | 5.211 | 3.880 | 3.711 | 3.927 | 5.389 |
| 26 | YLYAH-3Y | 5.081 | −12.366 | −13.437 | 1.211 | 1.974 | 2.543 | 1.770 | 1.293 | 2.015 | 3.047 |
| 27 | YLYG-1B- | 5.221 | −13.146 | −13.762 | 3.366 | 4.159 | 4.422 | 3.640 | 3.849 | 4.225 | 6.920 |
| 28 | YLYJ-1B- | 7.046 | −14.191 | −14.933 | 2.437 | 3.646 | 3.805 | 2.832 | 2.954 | 3.361 | 6.386 |
| 29 | YLYJ-3Y- | 7.129 | −11.306 | −12.011 | 1.693 | 2.941 | 2.902 | 2.125 | 2.120 | 2.616 | 5.325 |
| 30 | YLYLG-1Y | 4.145 | −10.161 | −11.209 | 1.751 | 3.393 | 3.197 | 2.432 | 2.615 | 2.820 | 5.270 |
| 31 | YLYLG-2P | 4.917 | −9.816 | −10.894 | 1.206 | 2.830 | 2.725 | 2.027 | 1.954 | 2.166 | 4.720 |
| 32 | YLYLG-3B | 1.713 | −5.016 | −5.411 | 0.694 | 1.633 | 1.502 | 1.035 | 1.325 | 1.462 | 3.000 |
| 33 | YLYLL-2P | 11.376 | −12.427 | −12.576 | 2.255 | 4.104 | 3.995 | 2.637 | 2.553 | 3.337 | 6.969 |
| 34 | YLYLL-3Y | 3.776 | −9.972 | −10.521 | 2.110 | 2.608 | 2.873 | 2.085 | 2.548 | 2.627 | 5.042 |
| 35 | YLYLS-3Y | 8.516 | −13.249 | −13.318 | 4.545 | 4.876 | 5.327 | 4.518 | 5.067 | 5.261 | 7.258 |
| 36 | YLYLT-1B | 0.8178 | −2.960 | −3.121 | 1.152 | 2.216 | 1.472 | 1.004 | 1.508 | 1.184 | 3.950 |
| 37 | YLYM-2P- | 4.229 | −9.784 | −10.325 | 2.200 | 3.838 | 3.510 | 2.411 | 2.517 | 3.225 | 7.041 |
| 38 | YLYM-3Y- | 9.595 | −11.998 | −13.632 | 9.286 | 10.532 | 9.860 | 8.470 | 8.911 | 10.315 | 13.188 |
| 39 | YLYQ-3Y- | 5.703 | −11.338 | -−11.497 | 3.413 | 5.245 | 5.209 | 4.237 | 4.286 | 4.586 | 7.003 |
| 40 | YXHC-2P- | 5.414 | −9.318 | −10.044 | 0.927 | 2.921 | 2.557 | 1.468 | 1.057 | 1.875 | 3.564 |
| 41 | YXHC-3Y- | 3.785 | −6.331 | −6.443 | 0.629 | 2.250 | 1.761 | 0.890 | 0.714 | 1.277 | 2.785 |
| 42 | YXHE-3Y- | 7.448 | −12.554 | −13.017 | 1.408 | 3.001 | 2.916 | 1.991 | 2.027 | 2.331 | 3.626 |
| 43 | YXHH-4P- | 4.146 | −7.190 | −7.823 | 1.489 | 3.134 | 2.699 | 1.641 | 2.019 | 2.422 | 3.886 |
| 44 | YXHH-5Y- | 1.169 | −4.428 | −4.999 | 0.776 | 2.014 | 1.722 | 0.936 | 1.147 | 1.385 | 2.123 |
| 45 | YXJD-1B- | 13.584 | −12.831 | −13.817 | 1.448 | 3.788 | 3.503 | 2.317 | 1.355 | 1.906 | 2.738 |
| 46 | YXJG-1B- | 8.095 | −12.168 | −12.948 | 4.461 | 5.314 | 5.800 | 3.790 | 4.271 | 4.217 | 3.939 |
| 47 | YXJG-2P- | 8.275 | −14.456 | −14.757 | 5.957 | 6.478 | 7.071 | 4.977 | 5.673 | 6.019 | 4.645 |
| 48 | YXJG-2Y- | 7.139 | −8.024 | −8.021 | 2.555 | 3.580 | 3.395 | 2.090 | 2.762 | 2.635 | 2.097 |
| 49 | YXJS-1B- | 3.232 | −5.876 | −7.096 | 3.057 | 4.304 | 3.958 | 2.767 | 3.232 | 3.143 | 3.279 |
| 50 | YXJS-2P- | 1.398 | −6.406 | −7.726 | 3.507 | 4.583 | 4.316 | 3.127 | 3.573 | 3.651 | 4.380 |
| 51 | YXJS-3Y- | 2.023 | −9.045 | −11.131 | 6.455 | 7.020 | 7.195 | 5.713 | 6.340 | 6.454 | 7.067 |
| 52 | YXJY-2P- | 6.745 | −13.415 | −14.076 | 6.390 | 6.633 | 7.330 | 5.788 | 6.713 | 6.687 | 6.308 |
| 53 | YXWA-1B- | 3.032 | −10.722 | −11.606 | 1.565 | 3.478 | 3.312 | 2.126 | 1.812 | 2.554 | 5.722 |
| 54 | YXWA-2P- | 3.849 | −11.433 | −11.928 | 1.998 | 4.234 | 3.940 | 2.726 | 2.581 | 3.286 | 6.886 |
| 55 | YXWA-3Y- | 3.691 | −10.166 | −10.769 | 1.498 | 3.014 | 2.833 | 1.897 | 1.992 | 2.482 | 5.688 |
| 56 | YXWL-3Y- | 6.866 | −11.809 | −12.999 | 4.796 | 6.804 | 6.521 | 4.595 | 5.518 | 6.028 | 6.522 |
| 57 | YXWPM-3Y | 11.687 | −13.920 | −13.797 | 3.787 | 5.867 | 5.530 | 4.394 | 3.867 | 4.440 | 7.183 |
| 58 | YXWTC-3Y | 4.572 | −12.768 | −13.474 | 2.318 | 3.820 | 4.011 | 2.774 | 3.022 | 3.221 | 4.938 |
| 59 | YXWYT-1B | 5.176 | −9.757 | −9.665 | 2.524 | 3.625 | 3.601 | 2.848 | 3.347 | 3.423 | 5.398 |
| 60 | YXWYT-2P | 9.422 | −14.990 | −14.68 | 3.148 | 4.451 | 4.367 | 3.483 | 4.161 | 4.463 | 7.601 |
| 61 | YXWYT-3Y | 9.597 | −9.580 | −9.540 | 3.957 | 5.142 | 4.543 | 4.104 | 5.11 | 5.166 | 7.861 |
| 62 | YXXD-1B- | 8.926 | −12.958 | −14.697 | 3.932 | 7.352 | 6.962 | 5.406 | 4.898 | 5.683 | 7.734 |
| 63 | YXXG-3B- | 10.785 | −13.687 | −14.008 | 3.191 | 6.628 | 6.001 | 4.682 | 3.700 | 4.736 | 7.450 |
| 64 | YXXK-3Y- | 8.344 | −13.101 | −13.555 | 3.496 | 4.092 | 4.608 | 3.897 | 3.888 | 4.111 | 4.099 |
| 65 | YXXN-3Y- | 11.209 | −14.005 | −13.96 | 6.944 | 8.575 | 8.543 | 7.266 | 8.061 | 8.472 | 8.848 |
| 66 | YXXP-1B- | 8.073 | −12.540 | −12.903 | 4.191 | 6.506 | 5.991 | 4.816 | 4.513 | 4.775 | 7.847 |
| 67 | YXXP-2P- | 4.331 | −10.691 | -12.049 | 2.109 | 4.086 | 3.970 | 2.620 | 2.240 | 2.617 | 5.250 |
| 68 | YXXQ-1B- | 5.579 | −13.046 | −14.070 | 4.666 | 5.336 | 5.988 | 4.494 | 5.167 | 5.136 | 5.479 |
| 69 | YXXQ-3Y- | 2.541 | −5.763 | −5.677 | 1.820 | 2.685 | 2.637 | 1.802 | 2.439 | 2.043 | 2.925 |
| 70 | YXXZ-3Y- | 6.831 | −11.894 | −13.483 | 3.342 | 4.887 | 5.311 | 3.863 | 3.617 | 4.007 | 4.538 |

**Table S3** Sensor properties and the number of Maca volatiles detected.

| No. | Sensor | Target | Reference | Number of maca volatiles detected |
| --- | --- | --- | --- | --- |
| 1 | LY/LG | Strong oxidizing gases | Chlorine, fluorine, nitrogen, oxygen compounds | 38 |
| 2 | LY2/G | Toxic gases | Ammonia, amines and hydrocarbon | 27 |
| 3 | LY2/AA | Organic compounds | Ethanol | 25 |
| 4 | LY2/gCT | Flammable gases | Propane and butane | 37 |
| 5 | LY2/gCTL | Toxic gases | Hydrogen sulfide | 29 |
| 6 | T30/1 | Organic compounds | Organic compounds | 41 |
| 7 | P10/1 | Combustible gases | Carbohydrate | 37 |
| 8 | P10/2 | Combustible gases | Methane | 34 |
| 9 | P40/1 | Strong oxidizing gases | Fluorine | 38 |
| 10 | T70/2 | Aromatic compounds | Toluene and xylene | 41 |
| 11 | PA/2 | Organic compounds and toxic gases | Ethanol, ammonia and amines | 40 |
| 12 | P30/1 | Combustible gases and organic compounds | Combustible hydrocarbon products | 34 |
| 13 | P40/2 | Strong oxidizing gases | Chlorine | 39 |
| 14 | P30/2 | Organic compounds | Ethanol and combustible products | 36 |
| 15 | T40/2 | Strong oxidizing gases | Chlorine, fluorine, nitrogen, oxygen compounds | 39 |
| 16 | T40/1 | Strong oxidizing gases | Fluorine | 34 |
| 17 | TA/2 | Organic compounds | Ethanol | 34 |

**Table S4** Volatile organic compounds of Maca detected by the GC-MS.

| Classification | Compound | Retention time | Matching degree (%) |
| --- | --- | --- | --- |
| | [Esters](http://www.so.com/link?url=http://dict.youdao.com/search?q=esters&keyfrom=hao360&q=酯类用英语怎么说&ts=1520139346&t=4bb49d49ed52202a7a8fa6ad857ca35) | | --- | | Ethyl Acetate * | 2.283 | 89 |
|  | Acetic acid, butyl ester * | 4.819 | 93 |
|  | Propanoic acid, 2-methyl-, butyl ester * | 8.341 | 93 |
|  | Hexanoic acid, ethyl ester * | 8.669 | 87 |
|  | Acetic acid, methyl ester * | 1.981 | 98 |
|  | Vinyl crotonate * | 6.030 | 86 |
|  | 4-Hexen-1-ol, (4E)-, acetate | 10.555 | 88 |
|  | Acetic acid, hexyl ester | 9.455 | 91 |
| Acids | Methyltartronic acid * | 2.623 | 97 |
|  | Acetic acid * | 12.356 | 97 |
|  | Butanoic acid * | 14.965 | 81 |
|  | Crotonic acid * | 16.776 | 86 |
|  | Hexanoic acid * | 17.658 | 96 |
|  | Octanoic acid | 20.048 | 86 |
|  | Hexanoic acid * | 17.658 | 89 |
|  | Butanoic acid, 2,2-dimethyl- * | 15.999 | 82 |
| Ketones | Acetoin * | 9.546 | 97 |
|  | 2,3-Butanedione * | 3.069 | 97 |
|  | 2,3-Pentanedione | 4.447 | 90 |
|  | 2-Nonanone * | 11.523 | 92 |
|  | 2-Undecanone | 14.674 | 92 |
|  | 5,9-Undecadien-2-one, 6,10-dimethyl- | 17.799 | 81 |
|  | 2-Pentanone, 3-methyl- | 6.982 | 83 |
|  | 5-Hepten-2-one, 6-methyl- * | 10.616 | 93 |
| Aldehydes | Butanal, 3-methyl-* | 2.484 | 96 |
|  | Hexanal * | 5.004 | 93 |
|  | Nonanal | 11.598 | 90 |
| Alkanes | Undecane | 5.614 | 95 |
|  | Heptadecane, 2,6-dimethyl- * | 8.057 | 94 |
|  | Tridecane* | 10.067 | 96 |
|  | Pentadecane * | 13.347 | 97 |
|  | Octane, 4-ethyl- | 3.433 | 85 |
|  | Dodecane, 2,6,11-trimethyl- * | 11.108 | 95 |
|  | Tridecane, 4-methyl- | 3.605 | 98 |
| Olefines | D-Limonene * | 7.804 | 89 |
|  | yclohexene, 1-methyl-4-(1-methylethenyl)-, (S)- * | 7.804 | 97 |
|  | 4-Hexen-1-ol, (E)- * | 11.892 | 91 |
| Indoles | 1-Iodo-2-methylundecane | 11.258 | 92 |
| Alcohols | 1-Octanol, 2-butyl-* | 11.805 | 87 |
|  | R-(-)-1,2-propanediol * | 14.464 | 98 |
|  | 1-Butanol * | 6.611 | 95 |
|  | 1-Hexanol * | 10.917 | 81 |
|  | 3-Hexanol, 2-methyl- * | 16.441 | 84 |
|  | Isopropyl Alcohol * | 9.546 | 80 |
|  | 2,3-Butanediol * | 14.176 | 96 |
|  | 2-Propanol, 1-methoxy- * | 13.766 | 92 |
| Aromatics | Benzaldehyde * | 13.551 | 96 |
|  | Benzoic acid, ethyl ester * | 15.559 | 95 |
|  | Acetic acid, phenylmethyl ester * | 16.328 | 92 |
|  | Benzeneacetic acid, ethyl ester | 17.033 | 92 |
|  | Phenylglyoxal * | 17.307 | 95 |
|  | Benzyl alcohol * | 18.036 | 92 |
|  | Benzaldehyde, 3-methoxy- | 18.286 | 95 |
|  | Benzeneacetonitrile * | 18.665 | 83 |
|  | Benzenamine, 2-methoxy-4-methyl- | 21.120 | 85 |
|  | (3-Methoxyphenyl)acetonitrile | 22.636 | 93 |
|  | Diethyl Phthalate * | 23.161 | 85 |
|  | Benzoic acid | 23.798 | 96 |
|  | Acetamide, N-(phenylmethyl)- * | 24.710 | 93 |
|  | Benzenemethanamine, N-(phenylmethylene)- * | 25.057 | 92 |
|  | N-Hydroxymethyl-2-phenylacetamide | 26.118 | 91 |
|  | p-Xylene | 6.423 | 85 |
|  | Benzene, (isothiocyanatomethyl)- * | 20.549 | 93 |
| Pyridines | 1-Benzyl-3-hydroxypicolinic acid inner salt | 24.464 | 86 |
| Pyrazines | Pyrazine, 2-ethyl-6-methyl- * | 11.432 | 80 |
|  | Pyrazine, trimethyl- | 11.736 | 94 |
|  | Pyrazine, 2,6-diethyl- * | 12.675 | 91 |
|  | Pyrazine, tetramethyl- * | 12.865 | 97 |
|  | Pyrazine, 2,3-dimethyl- | 10.711 | 91 |
| Furans | 4H-Pyran-4-one,2,3-dihydro-3,5-dihydroxy-6-methyl- | 22.034 | 83 |
|  | Furan, 2-pentyl- * | 8.596 | 93 |
|  | 2(3H)-Furanone, dihydro-5-pentyl- | 19.785 | 80 |
| Pyrroles | Ethanone, 1-(1H-pyrrol-2-yl)- * | 18.997 | 94 |
|  | 1H-Pyrrole, 1-(phenylmethyl)- | 19.592 | 85 |
| Heterocycles | Cyclohexasiloxane, dodecamethyl- * | 10.992 | 92 |
|  | Cyclopentasiloxane, decamethyl- * | 7.732 | 83 |
| Amides | Acetic anhydride * | 9.712 | 97 |
|  | N-Benzylformamide | 25.283 | 89 |
|  | Propanoyl chloride, 2-methyl- | 6.236 | 89 |
| Ethers | Methyl propargyl ether * | 4.017 | 84 |
| Pyrimidines | 4(1H)-Pyrimidinone, 6-hydroxy- | 20.985 | 84 |

*, detected in all samples.

**Table S5** Origin prediction by using the OPLS-DA model.

| Origin | Number of samples | Prediction | | | | Accuracy rate  (%) |
| --- | --- | --- | --- | --- | --- | --- |
|  |  | Huidong | Dali | Lijiang | Shangri-La |  |
| Huidong | 12 | 12 | 0 | 0 | 0 | 100.0 |
| Dali | 12 | 0 | 12 | 0 | 0 | 100.0 |
| Lijiang | 24 | 0 | 0 | 21 | 3 | 87.5 |
| Shangri-La | 47 | 0 | 0 | 1 | 46 | 97.9 |
| Total | 95 | 19 | 12 | 22 | 51 | 95.8 |

**Figure S1.** Verification of the electronic nose parameters. (a) Three color varieties of Maca. (b) Thirty Maca samples of six cities, twelve towns and three color varieties. B, black; P, purple; and Y, yellow.


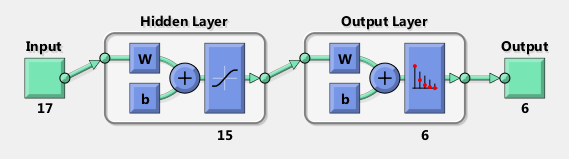


**Figure S2.** Scheme of the BP neural network.
